# “Opening the Box to Explore the Contents”: A study on the design elements of museum cultural and creative blind boxes based on consumer preferences – Taking the Macao Museum as an example

**DOI:** 10.1371/journal.pone.0344422

**Published:** 2026-03-06

**Authors:** Xuanrui Huang, Dequan Zhou, Qian Li, Haitong Ye, Haowen Ke, Anfeng Xu

**Affiliations:** 1 Faculty of Humanities and Arts, Macau University of Science and Technology, Taipa, Macau, China; 2 Tourism College, Hainan Tropical Ocean University, Sanya, Hainan, China; Politecnico di Torino, ITALY

## Abstract

This study adopts a consumer preference-driven approach and employs a mixed-methods research methodology to construct a systematic design decision-making model. By integrating literature review analysis, online review data mining, and screening, the study identifies preliminary key criteria for the design of museum-themed blind box cultural and creative products. Subsequently, the Fuzzy Delphi Method is applied to select the final 12 key design criteria, encompassing three dimensions: product value and experience, design aesthetics and craftsmanship, and cultural drivers and identity. The study uses museums in the Macau region as a case study and combines the Analytic Hierarchy Process to calculate the weighting ratios of each criterion. Based on the decision-making model and the weighting values of its internal elements, two design practices for blind box-style cultural and creative products were conducted, both of which utilized IPA as an auxiliary tool to ensure alignment with consumer preferences. The study also discusses the interdependent relationships among the various elements within the framework. The research findings provide theoretical support and optimization recommendations for museums in the development and design of blind box-style cultural and creative products, thereby promoting the better alignment of such products with consumer needs.

## 1. Introduction

With the rapid recovery of the urban economy and the steady growth of residents’ disposable income, there has been an expansion in demand for cultural consumption, driving the diversified development of the cultural and creative industries [1]. Among these, museum cultural and creative products, as representatives of cultural relics-related derivatives, have gradually become a key bridge between cultural dissemination and cultural consumption [2]. As a country with a rich historical and cultural foundation, China’s development path for museum cultural and creative products differs from the internationally prevalent market-oriented model [3,4], emphasizing the inheritance of cultural values and the function of soft power output [5]. In recent years, museum cultural and creative products have gradually been integrated into the national cultural consumption structure and industrial upgrading strategic layout. Since 2015, the central and local governments have successively introduced a number of encouraging policies covering cultural and creative conversion, intellectual property protection, digital technology application, and other aspects, highlighting that museum cultural and creative products have gradually developed in the direction of specialization, commercialization, and diversification [6,7]. Data from the National Cultural Heritage Administration shows that by 2020, the number of museum cultural and creative products in China had exceeded 124,000 varieties, with cumulative revenue surpassing 1.1 billion RMB. By 2022, the market size of museum cultural and creative products had exceeded 65 billion RMB, representing a year-on-year growth of 18.6% [8]. Museum cultural and creative products are increasingly becoming an important component of China’s cultural industry [9].In the context of the continuous diversification of cultural and creative product forms and the upgrading of consumer experiences, new types of museum cultural and creative products represented by blind boxes have rapidly emerged and gradually become an important medium connecting cultural expression and market consumption [10]. Such products typically draw inspiration from museum collections, historical figures, or cultural symbols, combining mechanisms such as “mystery packaging,” “limited edition releases,” and “series collection” to enhance product uncertainty and interactivity, thereby stimulating consumers’ curiosity and collecting motivation [10,11]. By integrating narrative storytelling, visual design, and interactive mechanisms, museum blind boxes establish an emotionally charged consumption experience chain between cultural translation and product commercialization.

Compared to traditional souvenir-style cultural and creative products, blind box formats offer greater fun, immersion, and social appeal. Their consumer base is increasingly characterized by a younger demographic, catering to young consumers’ multifaceted demands for self-expression, emotional projection, and cultural interaction [11,12]. The “surprise factor,” “cultural narrative,” and “social symbolism” [13] offered by blind box products align with the consumption psychology of Generation Z (those born between 1995 and 2009) in terms of “identity,” “expression,” and “participation.” The cultural and creative market is undergoing a profound transformation from a “display-oriented” to a “consumption-oriented” model [14].

The design elements of museum cultural and creative blind boxes are key factors influencing young consumers’ purchasing preferences. Existing research has focused on factors such as aesthetic value, cultural symbolism, regionally innovative design, and interactivity. Through diverse image designs, digital display methods, and the integration of cultural elements, these designs aim to stimulate users’ immersive experiences and participation motivation [10,12,15]. However, the current design approach for blind box cultural and creative products remains largely experience-driven, lacking systematic understanding and design criteria tailored to specific consumer groups, particularly young consumers. This limits the depth of response to consumer value perceptions and hinders the formation of precise innovation pathways for the products.

**Fig 1:**
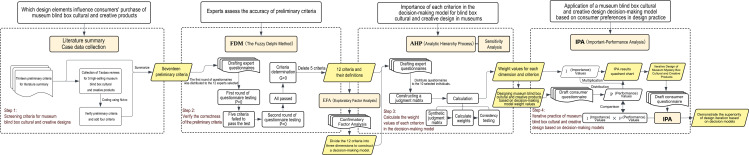
Research flowchart.

Current research on museum cultural and creative blind boxes primarily focuses on market trends or product analysis. While some studies touch on design aspects, there remains a lack of systematic integration regarding “design elements from the consumer’s perspective,” particularly in the areas of “design decision-making model construction” and “consumer recognition.” Effective evaluation frameworks and data validation pathways are insufficient, leading to a disconnect between theory and practice. Therefore, this study aims to construct a design evaluation framework for museum cultural and creative blind box products based on consumer preferences. By focusing on young consumers’ value perceptions, behavioral motivations, and design perceptions, the study identifies key design elements from the perspective of their purchasing intentions and constructs a decision-making model. Subsequently, the decision-making model is used for simulated design, and the created virtual cultural and creative products are presented to consumers for evaluation through renderings. This further validates that using the decision-making model to assist in museum blind box cultural and creative design can effectively improve consumer recognition and purchasing desire (Fig 1). It should be noted that the core mechanism of blind boxes’ “randomness,” including the probability settings for “hidden variants,” forms a crucial foundation of their product appeal. However, this study focuses on the essence of blind boxes as “cultural and creative products,” aiming to systematically analyze the design elements of their physical carriers and emotional content, rather than the probabilistic mechanisms of their business model. Therefore, the research treats “randomness” as a given premise that stimulates consumers’ motivation for exploration and collection. It primarily explores how, within this framework, specific design practices can optimize the product’s cultural value, aesthetic experience, and emotional connection, thereby more precisely responding to consumers’ deep-seated preferences. The contributions of this study can enhance the alignment between museum cultural and creative blind boxes and consumer preferences, providing cultural institutions and cultural and creative designers with more actionable, user-oriented guidelines for product development and optimization.

## 2. Literature review

### 2.1. Blind box

The origin of blind boxes can be traced back to Japanese commercial culture. As early as the 1960s, Japanese merchants introduced the “Fukubukuro” (lucky bag) sales model, which involved bundling various products into bags and clearly labeling them with a total value far exceeding their selling price, attracting consumers with the promise of a cost-effective surprise [16]. Subsequently, the emergence of “Gashapon Machines” further advanced this business model. These vending machines, specifically designed for toys, allowed consumers to insert coins and receive a capsule toy with a random style, without knowing the exact content before purchase, thereby enhancing the sense of uncertainty in the consumption experience.

Entering the 21st century, the blind box model deeply integrated with pop culture, gradually evolving into serialized products with uniform packaging, similar weight, but varying internal designs. This innovative format quickly gained popularity among younger generations. In 2016, the Chinese trendy toy company POP MART launched its first blind box series, accumulating a large number of loyal fans with its exquisite designs and unique purchasing experience [17,18]. The success of this business model also inspired the cultural and museum sector. In recent years, an increasing number of museums have developed culturally distinctive blind box products based on their collection themes or mascot designs, allowing traditional culture to reach the public in novel ways.

The appeal of blind boxes stems from their unique consumer psychological mechanisms. The uncertainty during the purchase process and the excitement of unboxing continually stimulate consumers’ curiosity [18,19]. Throughout the process from payment to unboxing, consumers experience emotions such as anticipation, tension, satisfaction, or disappointment, making this psychological fluctuation a key factor driving repeat purchases. At the same time, the strategy of selling products in sets successfully taps into people’s collecting instincts, as the desire to “complete the entire set” motivates consumers to continue investing. Additionally, the refined product designs and a moderate sense of “gambling” psychology further enhance the consumption experience, transforming blind boxes from mere toys into a new form of cultural consumption that combines emotional fulfillment and social interaction [20].

### 2.2. Value perception and emotional design theory

Value perception theory is a core theory in consumer behavior research, used to explain how consumers subjectively evaluate products or services during the purchasing decision process. Emotional design complements product design by introducing emotional value from a user experience perspective. By incorporating value perception theory and emotional design, this study provides a theoretical foundation for understanding how different design elements of cultural and creative products influence consumers’ purchasing intentions.

The concept of value perception theory first appeared in the theory of competitive advantage [21], which proposed that companies should build their own value chains when designing products to gain a competitive edge. Subsequent scholars divided value perception into four dimensions—emotional, social, quality, and price [22]—and established a stable evaluation framework. Domestic scholars have applied this theory to the context of museum cultural and creative products, defining six perceived value dimensions—quality, social, price, innovation, education, and experiential value—based on the positive and significant influence of innovation and experiential value on purchase intent. In the category of museum cultural and creative blind boxes, they analyzed consumer experiences from three aspects—emotional, social, and functional—to seek an appropriate framework [23].

Meanwhile, emotional design theory complements the emotional value of product design from a user experience perspective. Emotional design is divided into three levels: instinctive, behavioral, and reflective [24], which respectively address users’ first impressions of a product, their interactive experiences during use, and deeper meaningful connections. Museum cultural and creative products inherently possess emotional attributes due to their combination of functionality and cultural significance. Many domestic scholars, based on Norman’s theory, have divided museum cultural and creative design into three levels to reveal the mechanisms of emotional arousal. In the exploration of design elements for museum blind box cultural and creative products, it is necessary to establish visual impressions at the instinctive level, enhance interactive experiences at the behavioral level, and further evoke consumers’ emotions such as “surprise” and “joy” through mystery and randomness [25].

Therefore, through value perception theory and emotional design theory, this study jointly reveals the significant impact of emotions and value perception on consumer experience in cultural and creative blind box design, providing theoretical support for further exploration of how different design elements in cultural and creative products influence consumers’ purchasing intentions.

### 2.3. Design elements of museum cultural and creative products

The literature reviewed for this section was sampled based on specific criteria. Articles were required to include “blind box” and “design” in their titles or keywords and to be published in recent years, coinciding with the gradual mainstreaming of the blind box mechanism. Since blind box cultural and creative products under the theme of museums are relatively scarce, the keyword “museum” is not strictly required. The research team then conducted qualitative screening through manual literature review to identify whether new design elements were proposed. When multiple articles proposed identical or encompassing design elements, priority was given to those offering more comprehensive explanations or detailing a greater number of design elements. During the analysis and extraction of design elements from past literature, the focus was solely on the design-side attributes of blind boxes, such as physical characteristics and inherent product features, while explicitly excluding user-side experiential outcomes like surprise or mystery, which only manifest after consumer acquisition.

In existing literature, users’ perception of the value of museum cultural and creative blind box products is primarily reflected in the product’s functionality and sensory experience. For example, factors such as price acceptability, practicality, color coordination, aesthetic design, craftsmanship, and material quality form the basis of users’ initial judgments of the product [26,27]. These perceptions not only directly influence consumption decisions but also constitute the foundation of user experience. Additionally, some scholars have pointed out that consumers’ experiential feelings and social interactions further reinforce their preference for blind boxes. For instance, users may develop a strong affinity for a specific museum’s brand, and this emotional inclination often translates into purchasing intent [28]. In practical use, users engage in social interactions through activities such as unboxing shares and gifting [6], thereby enhancing emotional resonance and word-of-mouth dissemination [29]. Meanwhile, many studies emphasize the participatory interaction process between users and products, such as excavation-style cultural and creative blind boxes, which can effectively enhance user retention and stickiness.

Many scholars also focus on the value of blind box cultural and creative products in terms of cultural expression and dissemination. Through unified series theme designs, combined with original IP or innovative forms of creative expression [30], blind box cultural and creative products have acquired cultural carrying capacity. In addition, the cultural stories contained in blind boxes help to stimulate emotional connections among users and achieve cultural dissemination [31].

Therefore, this study focuses on the emerging product form of “museum cultural and creative blind boxes,” which has garnered significant attention in recent years. From a consumer perspective, it combines the core theoretical dimensions of “perceived value theory” and “emotional experience” to construct a theoretical framework for evaluating the influence of design elements of museum cultural and creative blind boxes on consumer purchase intentions. Although previous studies have preliminarily explored the design elements of such products, the findings remain scattered and lack systematic integration, particularly in terms of constructing a design element evaluation framework from the consumer’s perspective. The process of summarizing preliminary design criteria involved consolidating commonalities in how different authors described and interpreted various design elements, thereby forming a unified definition for each guideline. Throughout this process, elements were extracted and criteria refined without proceeding to categorize them into broader dimensions. A total of 14 evaluation criteria potentially influencing users’ value perception were identified, providing theoretical support for the expansion and refinement of the criteria system in future research (Table 1).

**Table 1:** Preliminary design criteria summarized from the literature.

Elements	Definition	References
E1 Price	Consumers’ assessment of product value for money and their acceptable price range	[26,27,32]
E2 Collection value	The collectible value of the product and the surprise experience brought by the unboxing process.	[29,30]
E3 Brand loyalty	The degree to which consumers like a specific museum brand or its thematic culture	[26,28]
E4 Social communication	Consumers’ sharing behavior with friends or on social media platforms during the process of purchasing, unboxing, gifting, etc.	[6,29,31]
E5 logistics speed	The time from purchase to receipt of blind box cultural and creative products	NEW
E6 Practical functions	The product is practical and functional in everyday life.	[26,31]
E7 Color coordination	The harmony between product colors and their visual appeal	[26,30]
E8 Product odor	Any scent (neutral, pleasant, or off-putting) that greets the user upon unboxing, influencing perceived freshness, authenticity, and overall emotional safety.	NEW
E9 Packaging quality	At no stage (survival, sale, after-sales service) should the cultural and creative products inside the museum blind box be damaged	NEW
E10 Manufacturing craftsmanship	The technical level and quality of the product are reflected in the manufacturing process.	[27,30]
E11 Aesthetic appeal	The aesthetic value and visual refinement embodied in the overall design of the product.	[26,30]
E12 Materials	The types of materials used in the product and their tactile and visual qualities, as well as other sensory experiences.	[26,30]
E13 Theme series	Whether to develop multiple series of products under a unified theme to enhance systematicity and continuity	[6,27,32]
E14 Image innovation	Whether it features original IP characters or employs innovative forms of expression to demonstrate creativity.	[26,30]
E15 Participatory interaction	Consumers can interact with blind box cultural and creative products.	[26,27]
E16 Cultural narrative	Products can tell cultural stories through design, evoking users’ emotional connections to history or culture.	[26,28,29,31]
E17 Cultural dissemination	The performance and potential of products in promoting cultural awareness and expanding cultural influence	[28,30,31]

## 3. Methods and data

### 3.1. Research design and methods

The construction of a design decision-making model for museum blind box cultural and creative products is diverse and complex, with its criteria first summarized using inductive analysis from relevant literature. Referring to the relevant literature in Section 2.3 of this paper, 14 preliminary criteria for museum blind box design decisions were inductively summarized, namely: price, added value, brand loyalty, social interaction, practical functionality, color coordination, manufacturing craftsmanship, aesthetic design, material selection, series theme, creative expression, participatory interaction, cultural narrative, and cultural dissemination. However, most literature differs in design criteria due to variations in empirical regional characteristics and subject matter, and most do not detail consumer needs, merely providing a general design framework. Since this paper focuses on a consumer preference-driven design decision-making model, the preliminary criteria summarized from the literature cannot be fully applied. The research team used the preliminary guidelines as a foundation and analyzed consumer preferences expressed in actual outstanding cases of museum blind box cultural and creative products in China to attempt to identify new guidelines [33].

Based on China’s largest online shopping platform, Taobao, and the sales data provided by Taobao regarding museum blind box cultural and creative products [34], the research team selected the five highest-selling museum blind box cultural and creative products: Guangdong Provincial Museum’s “Jade Carved Chicken Blind Box,” the Palace Museum’s “Palace Treasures Blind Box,” the Dunhuang Museum’s “Twenty-Four Solar Terms Blind Box,” the Henan Museum’s “Archaeology Blind Box,” and the Sanxingdui Museum’s “Sanxingdui Blind Box.” A total of 13,268 purchase reviews for these five blind box cultural and creative products were scraped from Taobao between April 1, 2023, and April 1, 2025. To ensure the representativeness of the scraped reviews, those with fewer than eight words (the median number of words in all reviews) were filtered out [35]. This process ultimately yielded 8,624 valid reviews for subsequent analysis. The research team assembled three trained coders, all holding master’s degrees in design studies with years of experience in product and packaging design. They demonstrated strong sensitivity to design elements in cultural and creative products. Prior to formal coding, coders underwent standardized training covering: familiarization with theoretical foundations such as perceived value theory and theory of planned behavior; Mastering text coding methodologies; and conducting pre-testing on a sample of reviews to ensure consistent interpretation of various design elements. through group discussions, the coders refined the definitions and established explicit inclusion/exclusion criteria. During this inductive process, new codes emerged from the data to capture unique aspects of blind box products. This process resulted in a standardized Coding Manual, which details the operational definitions, keywords, and anchor examples for each criterion (see S1 Appendix).Manual coding refined and supplemented the initial criteria (Table 2), adding three design elements to establish comprehensive design guidelines for museum blind box cultural and creative products. To assess coding quality, the study employed Holsti’s Coefficient to evaluate inter-coder reliability [36]. Using Excel’s RAND function to randomly select 10% of the total reviews (864 comments), three coders independently coded the samples. The resulting inter-coder reliability coefficient was 0.9429, indicating high consistency and reliability in the coding outcomes(see S1 Table in S2 Appendix).Subsequently, the FDM (Fuzzy Delphi Method) was employed to invite 12 experts from the cultural and creative design industry to validate the accuracy of all design criteria. Subsequently, Exploratory Factor Analysis (EFA) was used to categorize the precise design criteria into three dimensions. These three dimensions and 12 criteria were then subjected to Analytic Hierarchy Process (AHP) calculations. The resulting weightage ratios of the different criteria dimensions were used to guide the design practice of museum blind box cultural and creative products. Finally, after designing the 1.0 museum blind box cultural products, we distributed an online questionnaire to 238 blind box enthusiasts recruited through online channels using product renderings. Based on the survey results, an Importance-Performance Analysis (IPA) was conducted to iterate the design. The satisfaction levels of consumers after the iterative design were measured to demonstrate the superiority and accuracy of the decision-making model, providing reference value for future museum blind box cultural and creative design.

**Table 2:** Preliminary design criteria summarized from the literature.

	Criteria refinement	Code extraction	Original text
1	Brand loyalty; Packaging quality(New); logistics speed(New); Manufacturing craftsmanship; Cultural narrative; Color coordination	As a die-hard fan of the Guangdong Provincial Museum; The shipping speed was very fast; The packaging was well done; I really wanted to win the ginger and scallion chicken; The craftsmanship is quite intricate; The color is almost identical to the other two	Hahaha, as a die-hard fan of the Guangdong Provincial Museum, how could I not support the cultural and creative products of Guangning Jade Carved Chicken? The shipping speed was very fast, and the logistics were also efficient, so I received the package quickly. The packaging was well done, with no damage. I really wanted to win the ginger and scallion chicken (white-cut chicken), but ended up with the soy sauce chicken (soy sauce chicken), which is also great~ The craftsmanship is quite intricate, even the patterns are detailed. It’s heavier than I expected. To be honest, I only realized it was soy sauce chicken after checking the ingredients, hahaha, because the color is almost identical to the other two. I’ll definitely try again next time!
2	Image innovation; Manufacturing craftsmanship; Product odor(New); Packaging quality(New)	I must say that the design is truly creative; there is a dark spot on the left wing that cannot be wiped off; There is a strong odor, which is similar to the smell of a cheap, patterned phone case; I hope the quality control can be improved	I received the item, and I must say that the design is truly creative. However, there is a minor flaw: there is a dark spot on the left wing that cannot be wiped off. Additionally, there is a strong odor, which is similar to the smell of a cheap, patterned phone case—probably paint fumes—and it will take a few days of airing out to dissipate. I hope the quality control can be improved, with proper supervision, rather than just focusing on creativity. Material and finish: Some minor imperfections Product quality: Strong odor, needs to be aired out for a few days
3	Color coordination; Price; Cultural narrative; Image innovation	But this color is super cute; It’s cheaper than in stores; “open the door to good fortune”; The blind box is also very interesting	I couldn’t find the Phoenix Crown refrigerator magnet at the store, but this color is super cute, and with the Double 12 discount, it’s cheaper than in stores. The blind box is also very interesting, with a creative “open the door to good fortune” design that makes it worth buying.
4	Social communication; Collection value; Participatory interaction; Cultural narrative;	It was a gift; The whole process was quite interesting; Using the shovel and brush provided to dig it out was very interactive; The bronze artifact that was dug out felt quite substantial and had a nice texture; Can be used to teach them about history	It was a gift from a friend. The whole process was quite interesting. Using the shovel and brush provided to dig it out was very interactive. The bronze artifact that was dug out felt quite substantial and had a nice texture. It’s also suitable for children to play with and can be used to teach them about history. Adding a little water makes it easier to dig. I will definitely buy it again!
5	logistics speed(New); Packaging quality(New) Image innovation; Price	The shipping speed was pretty fast; The package was quite heavy; The idea of combining archaeological excavation with blind boxes is quite creative; if the cost-effectiveness could be improved	The shipping speed was pretty fast; I received it in just a few days. The package was quite heavy, feeling substantial in my hands. Archaeology has been quite popular lately, and the idea of combining archaeological excavation with blind boxes is quite creative. However, it feels a bit small, and if the cost-effectiveness could be improved, it would be even better. I look forward to more improvements in the future and hope that gift bags can be included.
6	Price; Cultural narrative; Materials Cultural dissemination	I spent 90 yuan; My luck was average; But the pottery is still pretty good; I hope to get a museum treasure next time.	How should I put it? I spent 90 yuan on a blind box and got a piece of primitive porcelain. My luck was average, but the pottery is still pretty good. I hope to get a museum treasure next time.

The questionnaires completed by the 12 cultural and creative design industry experts invited to this study and the 238 adult blind box purchasing enthusiasts recruited online were accompanied by an informed consent form provided to all participants at the outset. Consent was obtained prior to their commencement of the survey. Participants were required to read and acknowledge the informed consent form before proceeding with the questionnaire to ensure all participation was voluntary. All participants could contact the research team to withdraw at any stage of the study, including after completing the questionnaire. The research team would subsequently delete the corresponding data. Collected data was also securely stored by the research team to ensure the confidentiality and anonymity of survey participants. Communication and coordination regarding the invitation and questionnaire completion for the 12 cultural and creative design industry experts were conducted via email between February 10 and March 1, 2025. The 238 cultural and creative blind box enthusiasts were recruited online via “Weibo” and “Xiaohongshu” between February 10 and March 10, 2025. IPA questionnaire completion occurred on the “Wenjuanxing” platform during two sessions: March 27−28 and April 10−11, 2025. This project has been approved by the Clinical Research Ethics Review Committee of the University of Science and Technology of Macau/University Hospital (MUST/UH IRB) (Certificate Number: MUST-FA-2025005–3). Verbal informed consent was obtained from all participants prior to their commencement of the study.

### 3.2. Fuzzy Delphi method

Given that researchers may have differing definitions and interpretations of museum blind box cultural and creative products, this study utilized FDM to determine key criteria [37] in order to enhance the compatibility between the selected criteria and museum blind box cultural and creative products. The FDM employed in this study utilized “double triangle fuzzy numbers” and the “gray zone verification method” to determine whether expert perceptions exhibited consistent convergence effects [38,39]. FDM interviewed twelve professionals engaged in cultural and creative design, museum cultural and creative design education, and related fields, including museum cultural and creative designers, independent cultural and creative designers, and university faculty members. The experts involved in this study possess extensive experience in their respective professional disciplines, and their explicit or implicit knowledge on the subject has made significant contributions to this research. Additionally, since the focus of the study was on museum blind box cultural and creative products, it was hoped that the experts would appropriately consider the internal and external factors influencing such cultural and creative products in the design decision-making process. Meanwhile, researchers maintained communication with the experts throughout the interview phase to minimize any potential misunderstandings or personal biases [40]. The FDM calculation process follows the steps outlined by Zhu et al. [41], as detailed below:

Step (1): Perform statistical analysis on the “most conservative cognitive value” and “most optimistic cognitive value” provided by all experts for each element *i*, excluding outliers beyond “2 times standard deviations.” Then calculate the minimum value $C_{L}^{i}$, geometric mean $C_{M}^{i}$, maximum value $C_{U}^{i}$ in the remaining “most conservative cognitive value,” minimum value $O_{L}^{i}$, geometric mean value $O_{M}^{i}$, and maximum value of the “most optimistic perceived values”.

Step (2): Based on the calculation results from Step (1), compute the triangular fuzzy number $C^{i}=\left(C_{L}^{i},C_{M}^{i},C_{U}^{i}\right)$ for the “most conservative cognition” and the triangular fuzzy number $O^{i}=\left(O_{L}^{i},O_{M}^{i},O_{U}^{i}\right)$ for the “most optimistic cognition” of each evaluation element *i*.

Step (3): Use triangular fuzzy numerical deviation tests to determine whether expert opinions are consistent. If there is no overlap between the two triangular fuzzy numbers, that is, $C_{U}^{i}\leq O_{L}^{i}$, it indicates that the opinion interval value of each expert has a consensus section and that the opinion tends to be within this consensus section; therefore, the “consensus value” $G_{U}^{i}$ of this evaluation element *i* can be calculated by equation.


$$G_{U}^{i}=\frac{C_{M}^{i}+O_{M}^{i}}{\text{2}}$$

If there is an overlap between the two triangular fuzzy numbers, that is, $C_{U}^{i}>O_{L}^{i}$, and the gray area $Z^{i}=C_{U}^{i}-O_{L}^{i}$ of the fuzzy relationship is smaller than the range $M^{i}=O_{M}^{i}-C_{M}^{i}$ between the “geometric mean of optimistic cognition” and the “geometric mean of conservative cognition” for the expert evaluation criterion, it indicates that although there is no consensus section for each expert’s opinion interval value, the two experts who gave extreme opinions (the most conservative expert of the optimistic cognition and the most optimistic expert of the conservative cognition) do not differ much from other experts in opinion. Then the “consensus value” $G_{U}^{i}$ of this evaluation element *i* can be calculated by equation.


$$G_{M}^{i}=\frac{O_{M}^{i}\times C_{U}^{i}-O_{L}^{i}\times C_{M}^{i}}{\left(O_{M}^{i}-O_{L}^{i}\right)+\left(C_{U}^{i}-C_{M}^{i}\right)}$$

If $C_{U}^{i}>O_{L}^{i}$ and $Z^{i}=C_{U}^{i}-O_{L}^{i}$ are larger than $M^{i}=O_{M}^{i}-C_{M}^{i}$, it indicates that no consensus exists within the range of opinions provided by each expert. Furthermore, the two experts presenting extreme viewpoints—the most conservative optimistic expert and the conservative optimistic expert—diverge significantly from the opinions of other experts, resulting in substantial disagreement. Therefore, it is necessary to conduct a new round of questionnaires and repeat Steps (1) through (3) until all evaluation items converge and corresponding “consensus values” are obtained.

In the first round of the FDM expert questionnaire, E4, E8, E9, E11, and E15 from the first round of the FDM expert questionnaire failed validation (see sheet 1 in S3 Appendix). This indicates that experts did not maintain a high level of agreement regarding the importance of these five criteria. Steps 1–3 must be repeated. After discussion and revision, the five criteria proceeded to the second round of FDM (see sheet 2 in S3 Appendix). In the second round of the FDM expert questionnaire, all criteria passed the validation test (Table 3).

**Table 3:** The FDM results.

Elements	Conservative		Optimistic		Geometric		Verification	Consensus	Achieved the threshold?	Key Criteria
	Value C		Value O		Mean M					
	CL	CU	OL	OU	Cm	Om	M—Z	G		
E1 Price	5	8	7	9	6.15	7.98	0.83	7.35	YES	C1
E2 Collection value	5	7	6	8	5.90	7.12	0.23	6.50	YES	C2
E3 Brand loyalty	4	8	6	9	6.02	8.12	0.10	7.04	YES	C3
E4 Social communication	3	6	5	7	4.59	5.96	0.37	5.40	NO	
E5 logistics speed	2	6	4	8	3.28	5.37	0.09	4.67	NO	
E6 Practical functions	2	6	5	8	3.63	5.84	1.22	5.26	NO	
E7 Color coordination	4	7	6	8	5.45	7.23	0.78	6.44	YES	C4
E8 Product odor	2	5	4	7	3.46	5.60	1.14	4.51	NO	
E9 Packaging quality	2	8	6	9	5.63	7.79	0.16	6.86	NO	
E10 Manufacturing craftsmanship	2	8	6	9	5.63	7.79	0.16	6.86	YES	C5
E11 Aesthetic appeal	7	8	8	9	7.40	8.40	1.00	8.00	YES	C6
E12 Materials	5	7	6	9	5.61	7.02	0.41	6.42	YES	C7
E13 Theme series	4	7	6	9	5.85	7.71	0.87	6.60	YES	C8
E14 Image innovation	3	7	6	8	5.42	7.12	0.70	6.41	YES	C9
E15 Participatory interaction	4	8	6	9	5.73	7.78	0.05	6.88	YES	C10
E16 Cultural narrative	5	8	7	9	6.57	7.79	0.22	7.36	YES	C11
E17 Cultural dissemination	5	8	7	8	6.12	7.48	0.37	7.20	YES	C12

**Fig 2:**
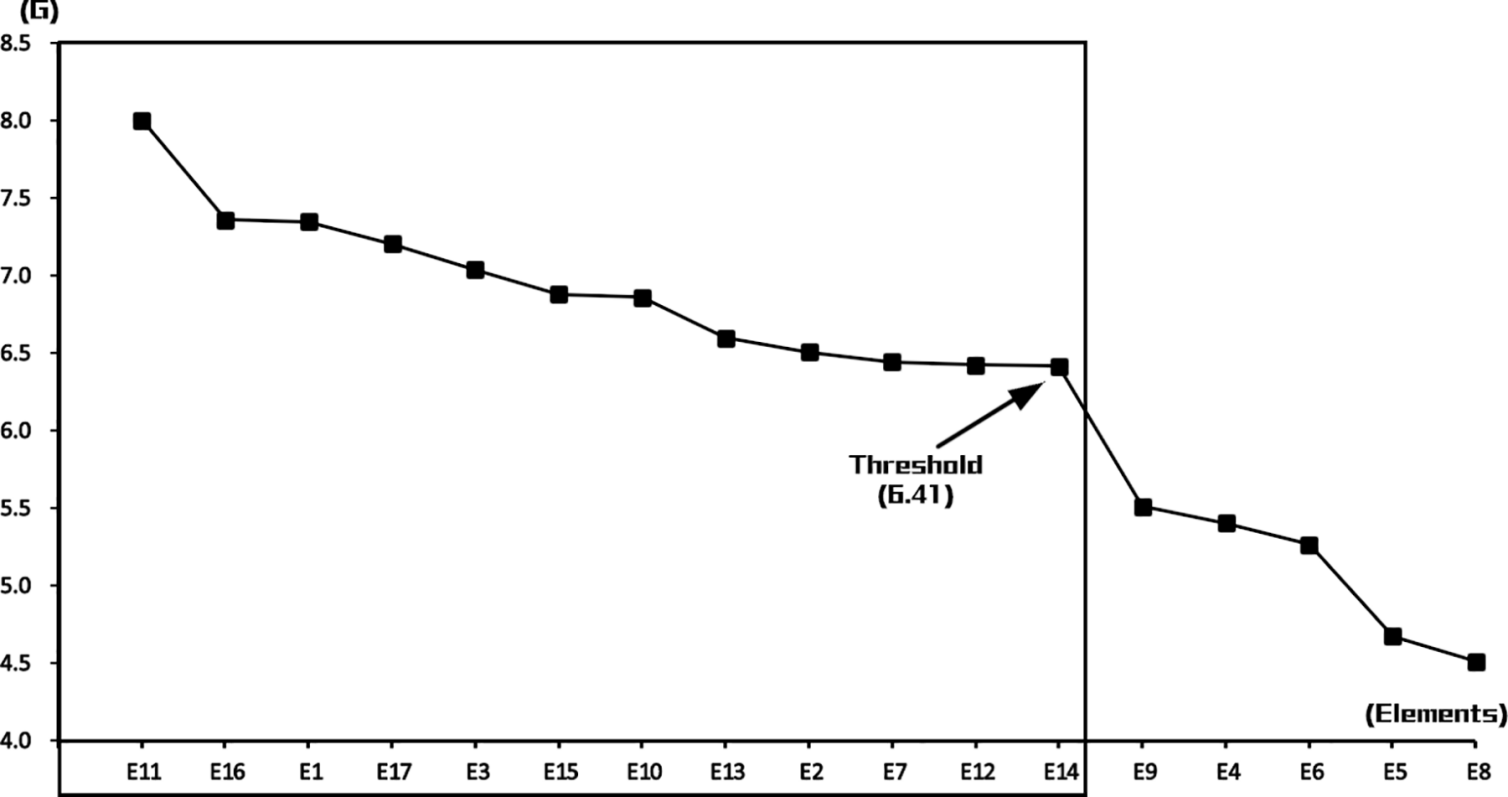
Scatter plot of expert consensus values.

The threshold for the expert consensus analysis was determined using the steep slope decision rule. Consensus values (G) for all elements were sorted in descending order to generate a line chart [42]. As shown in Fig 2, between E14 and E9, the line exhibits a pronounced “cliff-like” drop — the steepest gradient segment. This abrupt decline indicates substantial divergence in expert consensus for elements beyond E14, warranting their exclusion. Accordingly, the threshold was set at 6.41, corresponding to the G value of E14(see sheet 3 in S3 Appendix). Finally, the analysis revealed that the decision-making model incorporates 12 key criteria (see sheet 4 in S3 Appendix).

### 3.3.. Exploratory factor analysis

Exploratory Factor Analysis (EFA) was employed to achieve a more scientific organization and understanding of these design criteria. EFA is a statistical analysis method used to identify latent structures within data, simplifying multiple observed variables into a few factors to reveal the intrinsic relationships among variables [43]. In this study, the research team developed a questionnaire based on the 12 design criteria and physical artifacts, collecting a total of 211 valid questionnaire responses [44]. The data reliability and validity are presented in Table 4. Through EFA analysis of the 12 design criteria, these criteria were successfully categorized into three main dimensions: Product Value and Experience (D1), Design Aesthetics and Craftsmanship (D2), and Cultural Driven and Identity (D3). Each dimension represents a core theme in the design of museum cultural and creative blind boxes (Table 5).

**Table 4:** EFA questionnaire validity analysis.

Type	Observed value	Significance test results
KMO sampling adequacy measure	KMO Value	0.812
Bartlett’s sphericity test	Approximate Chi-squared value	606.263
	degree of freedom	66
	significance level	0.000

**Table 5:** EFA results.

Criteria		Factor proportion (%)		
		D1 (28.543)	D2 (51.952)	D3 (71.062)
C1	Price	0.846		
C2	Collection value			0.878
C3	Brand loyalty	0.829		
C4	Color coordination		0.842	
C5	manufacturing craftsmanship		0.826	
C6	Aesthetic appeal		0.842	
C7	Materials		0.856	
C8	Theme series	0.754		
C9	Image innovation	0.840		
C10	Participatory interaction	0.824		
C11	Cultural narrative			0.834
C12	Cultural dissemination			0.820

### 3.4. Confirmatory factor analysis

**Fig 3:**
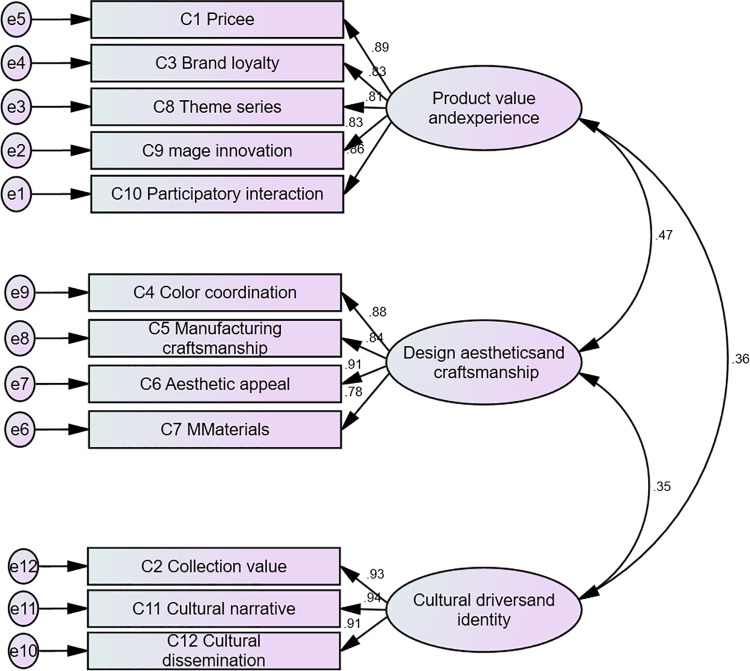
Confirmatory factor analysis model.

Confirmatory factor analysis (CFA) was employed to validate the fit of the three-factor model obtained through exploratory factor analysis (EFA). To ensure the model’s validity and reliability, CFA model fit tests were first conducted (Fig 3). The model fit results (see S2 Table in S2 Appendix) indicate excellent fit across all metrics: the CMIN/DF ratio of 1.933 demonstrates outstanding fit quality; the RMSEA value of 0.067 indicates the model falls within the excellent fit range; The SRMR value of 0.0049 indicates minimal residuals and favorable fit; TLI and CFI values of 0.971 and 0.977, respectively, further validate the model’s strong fit [45].

After completing the basic fit tests for the CFA model, we further examined the convergent validity and composite reliability (CR) of the scale. S4 Table in S2 Appendix shows that, based on the CFA model, the AVE values for all dimensions exceeded 0.5, and the CR values all exceeded 0.7, indicating that all measurement dimensions possess good convergent validity and composite reliability.

Additionally, discriminant validity was assessed using the HTMT method (see S3 Table in S2 Appendix). Results indicate that HTMT values for all dimensions were below 0.85, confirming good discrimination between constructs and ensuring clear differentiation across the scale’s dimensions [46].

### 3.5. Criterion priority analysis based on AHP

After screening, three dimensions (D1-D3) and 12 criteria (C1-C12) were identified, as shown in Table 5, forming a multi-attribute decision-making model. The Analytic Hierarchy Process (AHP) was employed to assign weights to the importance of the blind box cultural and creative design decision-making model, with 10 consumers completing a questionnaire [47]. The AHP method can both calculate criterion weights and rank alternative options [48]. These 10 consumers were recruited online by the research team as seasoned blind box cultural and creative product consumers who had purchased 15 or more such products, possessing a certain level of insight and familiarity with blind box cultural and creative products, and ensuring they had a basic understanding of the AHP questionnaire content. The AHP method determines the relative importance between different levels and elements through pairwise comparisons. Based on these comparison results, a judgment matrix is constructed. After the matrix is constructed, mathematical calculations are performed to determine the eigenvalues and eigenvectors of the judgment matrix, and consistency checks are conducted to ensure the reasonableness and reliability of the comparison results (see S4 Appendix). Finally, the criteria are ranked based on the weight values [43,49].

#### 3.5.1. Calculation of weights and consistency check of judgment matrix.

(1) Constructing a judgment matrix: Each decision maker assigns different levels of importance to each factor, and this assessment of importance is based on the decision maker’s own perceptions. To quantify these qualitative perceptions, a 9-point scale was used (Table 6). These values were determined based on the decision makers’ intuitive judgments during the qualitative analysis.

**Table 6:** Score meaning.

Score	Definition
1	Both criteria are equally important.
3	Comparison between the two criteria: the former is moderately important than the latter.
5	Comparison between the two criteria: the former is strongly important than the latter.
7	Comparison between the two criteria: the former is very strongly important than the latter.
9	Comparison between the two criteria: the former is extremely important than the latter.
2.4.6.8	The median value between the above adjacent judgments
Countdown	If the importance ratio of criterion A_i_ to criterion A_j_ is A_ij_, then the importance ratio of criterion A_j_ to criterion A_i_ is A_ji_ = 1/A_ij_, (i, j = 1,2,... n)


$${\text{A}}_{\text{n}\times \text{n}}=[\begin{array}{cccc} {\text{a}}_{\text{11}} & {\text{a}}_{\text{12}} & {\text{a}}_{\text{1}..} & {\text{a}}_{\text{1n}}\\ {\text{a}}_{\text{21}} & {\text{a}}_{\text{22}} & {\text{a}}_{\text{2}..} & {\text{a}}_{\text{2n}}\\ {\text{a}}_{..} & {\text{a}}_{..} & {\text{a}}_{..} & {\text{a}}_{..}\\ {\text{a}}_{\text{n1}} & {\text{a}}_{\text{n2}} & {\text{a}}_{\text{n}..} & {\text{a}}_{\text{nn}} \end{array}]$$

(2) Merge the ten consumer judgment matrices to obtain a single composite matrix.

$$\overset{―}{\text{A}}=(\prod_{k=\text{1}}^{m}a_{ij}^{k}{)}^{\frac{\text{1}}{m}}$$

(3) Calculate the relative weights of the synthetic judgment matrix.

$${\text{W}}_{i}=\frac{(\prod_{j=\text{1}}^{n}a_{ij}{)}^{\frac{\text{1}}{n}}}{\sum_{i=\text{1}}^{n}(\prod_{j=\text{1}}^{n}a_{ij}{)}^{\frac{\text{1}}{n}}}\;,\;i=\text{1},\text{2},\text{3}..,n$$

(4) Matrix consistency test

Since the judgment matrix is derived from the decision-makers’ subjective judgments, inconsistencies may arise when different decision-makers make pairwise comparisons. Therefore, a consistency index (CI) is needed to assess consistency.


$$\begin{array}{c} \text{CI}=\frac{\lambda_{max}-n}{\left(n-\text{1}\right)},\lambda_{max}=\sum {\mathrm{\backslash nolimits}}_{i=\text{1}}^{n}\frac{{\left[\overset{―}{\text{A}}W\right]}_{i}}{{nW}_{i}} \end{array}$$

In addition, the consistency ratio (CR) is also used to evaluate whether the judgment matrix passes the consistency test. The random index (RI) represents the average consistency index. When CR = 0, the judgment matrix is considered to be completely consistent. When CR < 0.1, the consistency of the judgment matrix is considered acceptable; when CR > 0.1, the judgment matrix does not meet the consistency requirements [50].

#### 3.5.2. Calculation results.

Using the aforementioned calculation methods and steps, the maximum eigenvalue, eigenvector, consistency index (CI), and consistency ratio (CR) were calculated for each judgment matrix. Table 7 lists the data for each indicator in detail, and Table 8 lists the weights of each criterion in the museum blind box cultural and creative design decision-making model in detail.

**Table 7:** Layered index summary.

	D	D1-C	D2-C	D3-C
**λ**_**max**_	3.0885	5.1727	4.0410	3.0723
**CI**	0.0442	0.0432	0.0136	0.0361
**RI**	0.58	1.12	0.90	0.58
**CR**	0.0763	0.0385	0.0152	0.0623
**Results**	pass	pass	pass	pass

**Table 8:** Weighting table for each criterion in the decision-making model for museum blind box cultural and creative design.

Dimensions (D)	Weight (W)	Rank	Criteria (C)	Relative Weigh (RW)	Gross weight (GW)	Rank
Product value and experience (D1)	0.1864	3	C1 Price	0.3259	0.0608	8
			C3 Brand loyalty	0.1476	0.0275	11
			C8 Theme series	0.2781	0.0518	9
			C9 Image innovation	0.2002	0.0373	10
			C10 Participatory interaction	0.0482	0.0090	12
Design aesthetics and craftsmanship (D2)	0.4816	1	C4 Color coordination	0.2514	0.1211	3
			C5 Manufacturing craftsmanship	0.3295	0.1032	5
			C6 Aesthetic appeal	0.2142	0.1587	1
			C7 Materials	0.2048	0.0986	6
Cultural drivers and identity (D3)	0.3320	2	C2 Collection value	0.2068	0.0687	7
			C11 Cultural narrative	0.4370	0.1451	2
			C12 Cultural dissemination	0.3561	0.1182	4

### 3.6. Sensitivity analysis

The AHP employed in this study involved only ten participants. Decision outcomes derived from small-sample AHPs are susceptible to influence from multiple social environmental factors, thereby compromising the applicability of the results [51]. Therefore, sensitivity analysis was conducted to assess the robustness of the AHP findings. Given that the AHP judgments are based on the subjective perceptions of a specific group of experts and consumers, it is crucial to examine whether the final ranking of the design criteria (C1–C12) fluctuates significantly when the weights of the three core dimensions (D1–D3) are altered. This analysis simulates scenarios where the weight of each dimension varies from 0 to 1, while the local weights of the sub-criteria within each dimension remain constant [52].

**Fig 4:**
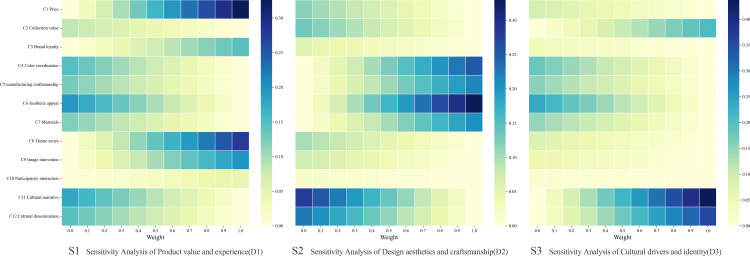
Results of the sensitivity analysis.

The results of the sensitivity (Fig 4) analysis respectively correspond to the weight changes in the dimensions of product value and experience (D1), design aesthetics and craftsmanship (D2), and cultural drive and identity recognition (D3).

S1 illustrates the sensitivity variations within the Product value and experience (D1) dimension, where the model exhibits a specific local sensitivity. As the weight of D1 increases, the global weight of Price (C1) demonstrates a steep upward trajectory. The analysis reveals that if decision-making preferences shift significantly towards “value-for-money”—specifically when the weight of D1 exceeds 0.4—Price (C1) has the potential to surpass aesthetic factors and become the dominant criterion. This implies that while the general model prioritizes aesthetics, cost control remains a critical determinant for price-sensitive consumer segments.

S2 demonstrates the high robustness of the Design aesthetics and craftsmanship (D2) dimension. Despite the variation in the weight of D2, the top-ranked criterion, Aesthetic appeal (C6), consistently maintains its leading position relative to other factors. Concurrently, Manufacturing craftsmanship (C5) exhibits a stable performance trajectory. This stability confirms that “visual attractiveness” constitutes the core competitiveness of museum blind box products and remains largely unaffected by fluctuations in consumer preferences.

S3 reveals a competitive yet balanced relationship between cultural content and visual form within the Cultural drivers and identity (D3) dimension. As the weight of D3 increases, the importance of both Cultural narrative (C11) and Cultural dissemination (C12) rises steadily. Notably, Cultural narrative (C11) consistently remains within the top tier of the ranking across most scenarios, reinforcing the finding that storytelling serves as a stable and essential component of the product’s value proposition.

In summary, the sensitivity analysis validates the reliability of the decision-making model proposed in this study. While Price (C1) exhibits sensitivity to specific value-oriented scenarios, the core design elements—Aesthetic appeal (C6) and Cultural narrative (C11)—exhibit high stability. This indicates that the weighting results derived in Section 3.5 are robust and can effectively guide the subsequent design practice for the Macau Museum blind box series.

## 4. Using decision models to guide design practice

The Macau Museum is committed to “advancing cultural heritage preservation, protecting cultural heritage, and promoting Macau’s unique blend of Chinese and Western cultures through an interdisciplinary international perspective and humanistic concern. It aims to foster connections between art and daily life, and explore the boundless possibilities of art” [53]. Therefore, the cultural and creative products currently available for sale at the museum possess profound cultural significance and artistic value. Inspired by the museum’s collection resources, these products incorporate creative design and elements of lifestyle aesthetics, resulting in five series comprising 17 distinctive cultural and creative products, such as metal filigree bookmarks, file folders, ceramic coasters, washi tape, and cotton handkerchiefs. Research into the names, era, product symbolism, materials, and prices, it was found that the existing cultural and creative products are reinterpreted based on elements such as the colors of historical artifacts, traditional Chinese and Western patterns, traditional customs and festivals, the background stories of historical districts and Macau’s development history, and the harmonious blend of elegance and practicality. The aim is to breathe new life into artifacts through modern products, allowing consumers to appreciate Macau’s history and culture while using them [54]. The sale of cultural and creative products began in 2020, with materials primarily sourced from eco-friendly plastics, ceramics, and pure cotton, priced between 15 and 65 MOP. By analyzing and summarizing the information on existing cultural and creative products, we can contribute to the design of Macau Museum’s blind box cultural and creative products.

### 4.1. First round of design

This blind box cultural and creative design scheme takes “Macao’s historical heritage buildings” as its core creative theme, aligning with the cultural and creative philosophy of the Macao Museum, which emphasizes “promoting the diversity of Chinese and Western cultures and bridging art with daily life.” Using an AHP model to establish design element weighting criteria, combined with field research findings and online user preference data, the design achieves cultural depth, visual distinctiveness, and practical cultural and creative functionality [55].

**Fig 5:**
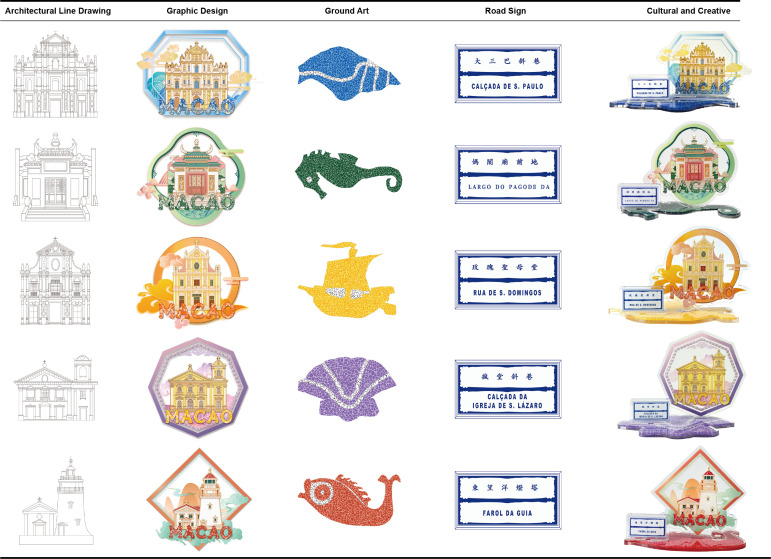
Macau Museum blind box cultural and creative product design first edition.

The design is based on the rankings of tourist check-in locations from Macau’s official tourism platform data [56], selecting five historical heritage buildings in Macau that are well-known to the public and representative of cultural significance (the Ruins of St. Paul’s, A-Ma Temple, Our Lady of the Rosary Church, East Ocean Lighthouse, and St. Anthony’s Church) [57]. These buildings are reimagined with cultural creativity and presented through modern aesthetic interpretations (Fig 5).

At the theoretical application level, this study employs Perceived Value Theory and Emotional Design Theory as frameworks to guide specific design decisions. Perceived Value Theory emphasizes consumers’ comprehensive evaluation of a product’s functional, emotional, and social value. Therefore, the design prioritizes price rationality (C1) and collectible value (C2), ensuring the product meets consumer value expectations in terms of affordability and scarcity. The three levels of affective design theory—instinctive, behavioral, and reflective—correspond respectively to: – Visual appeal through color coordination (C4) and aesthetic form (C6), – Practical functional experience via participatory interaction (C10), – And deep meaning connections through cultural narrative (C11) and cultural communication (C12). This approach ensures the product not only elicits visual appeal but also strengthens emotional resonance and cultural identity during use.

Then consideration of consumer focus areas identified through AHP, including: Aesthetic appeal (C6): The patterns build upon the original architectural structure and proportions, incorporating hand-drawn illustrative elements to enhance approachability. The composition integrates traditional Chinese garden landscape techniques such as framed views and borrowed scenery, creating visual depth and layering. Traditional motifs like auspicious clouds are used to convey blessings and good fortune; Cultural narrative (C11): Each blind box product is accompanied by the Portuguese street name corresponding to the historical building and Macau’s distinctive ground mosaic art patterns, reinforcing the historical story background and the fusion of Chinese and Western cultures; Color coordination (C4): By extracting the original building’s color palette and supplementing it with complementary colors, a harmonious and distinctive visual effect is achieved; Materials (C7): Material choices continue the use of eco-friendly acrylic commonly found in Macau Museum cultural and creative products; Collection value (C2): The three-dimensional stand design combines various Macau elements in a systematic manner, encouraging series collection. This series of Macau Museum blind box cultural and creative products can be used as souvenirs, with practical applications such as desk ornaments and refrigerator magnets, and strong static aesthetic appeal (Table 9).

**Table 9:** Weighting table for each criterion in the decision-making model for museum blind box cultural and creative design.

Dimensions	Implementation strategy
Historical building selection	Precise selection of locations based on visitor data to enhance cognitive foundation
Color expression	Based on the original colours of the buildings, supplemented by visual optimization
Integration of cultural stories	Each blind box contains cultural symbols (street signs, mosaic patterns)
Collectible and combinable	Can be combined to form small landscapes of Macau’s cultural landmarks, enhancing their collectible value
User perception matching	Strongly correlated with elements with high AHP scores

Aligning with the core mission of the Macau Museum “cultural revitalization and connection to daily life “this initiative fully incorporates the weighting factors of the AHP design methodology. The content highlights the unique cultural characteristics of Macau, resulting in an innovative series of architectural heritage-themed blind box cultural and creative products that blend tourist memorabilia with cultural dissemination.

### 4.2. Consumer satisfaction with the first round of designs

**Fig 6:**
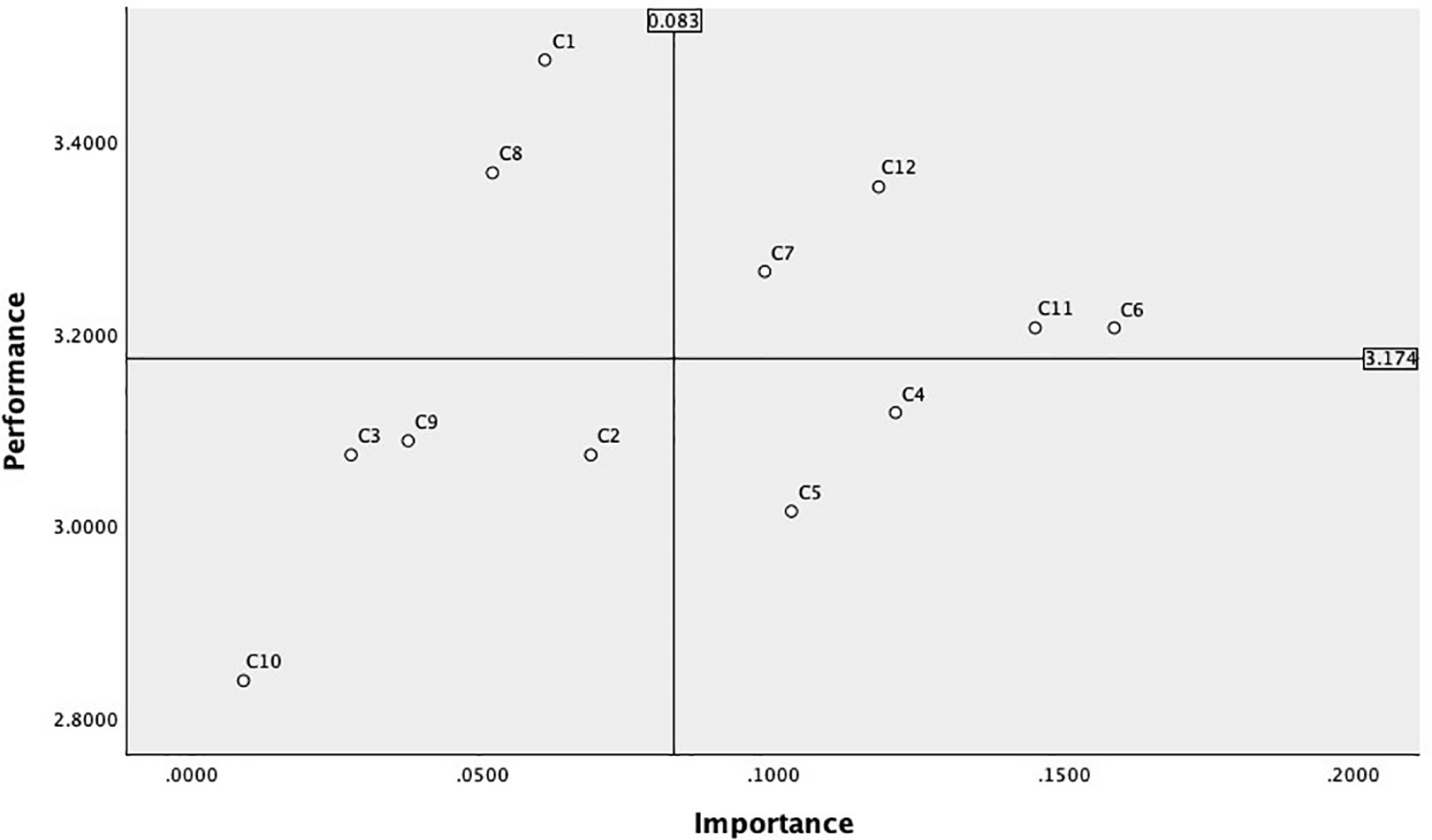
Macau Museum blind box cultural and creative product design first edition IPA results.

We recruited 238 enthusiasts of museum cultural and creative products through online recruitment. Selected participants must be high-quality consumers in both museum visits and cultural and creative product purchases. They should visit museums at least once every three months on average, with each museum cultural and creative product purchase amounting to 101 MOP or more. Demographic details are as shown in Table 10 [58,59]. A questionnaire was distributed to the 238 consumers, who were asked to observe virtual product renderings of cultural and creative products simulated in museum stores and 1–5 score 12 criteria based on their own experiences. The reliability and validity analysis of consumer rating data yielded a KMO value of 0.947 and a P-value of 0.000. The cumulative variance explained of 67% indicates that the data possesses high reliability (see S5 Table in S2 Appendix). This data was combined with AHP design element weights in SPSS 26 software to create a satisfaction score for the “Aocheng Xiaozhu—Architectural Signage Design” blind box cultural and creative product. Combining the AHP design element weights with the SPSS 26 software, a ‘importance’ and “satisfaction” matrix for the 12 elements of museum blind box cultural and creative products was created (Fig 6). The vertical axis represents “satisfaction,” and the horizontal axis represents “importance.” The coordinate system origin for constructing the IPA chart is based on the weighted average score (0.083) and the satisfaction average score (3.174) [60].

**Table 10:** Personal characteristics of the participants.

**Respondents (n = 238)**	
**Gender**	
Male	136
Female	102
**Country**	
China	163
East Asian and Northeast Asian countries (South Korea, Japan, Russia, etc.)	28
Southeast Asian countries (such as Thailand, Malaysia, Singapore, etc.)	42
European and American countries (such as the United States, the United Kingdom, Germany, France, etc.)	5
Other countries/regions	0
**Age**	
Under 18	0
18-30	195
31-40	29
41-50	12
Over 51	2
**Number of museum visits**	
On average once a month or more	96
On average once every three months	142
On average once every six months	0
On average once a year or less	0
**Amount spent on museum cultural and creative products**	
Average transaction amount of 100 MOP or less	0
Average transaction amount of 101–200 MOP	57
Average transaction amount of 201–500 MOP	178
Average transaction amount of 501–1000 MOP or more	3

The lower right quadrant of the matrix represents the improvement area, indicating that consumers place great importance on the Color coordination (C4) and manufacturing process (C5) of blind box cultural and creative products, but the current performance of such products falls short of expectations. Possible reasons include low color recognition with the theme of Macau’s architectural heritage, overly complex color combinations leading to visual fatigue, rough product manufacturing process, and product quality failing to meet expectations. In improving the design strategy, the contrast and coordination between the main architectural colors and auxiliary elements can be enhanced, and Macau-specific color schemes can be introduced (such as the pale yellow of the Rose Church, the blue-green and pale red of the brick walls of the A-Ma Temple, and the blue of Portuguese bricks); Optimize material selection and production precision, such as surface treatment and detail texture processing. These two elements are the weak points of the design of Macau Museum’s blind box cultural and creative products and deserve further attention.

The lower-left quadrant of the matrix represents the opportunity zone, where consumers place relatively low importance on the elements in this area and have low satisfaction levels. In terms of Collectible value (C2), Brand loyalty (C3), Image innovation (C9), and Participatory interaction (C10), consumers’ understanding of these concepts remains in a “blank zone,” offering significant potential for improvement. If designed appropriately, these elements can differentiate the product from others and establish its design advantages. Building brand and IP imagery and increasing product interactivity can serve as improvement methods to enhance consumer satisfaction with these aspects, laying the foundation for future product differentiation and user retention. The elements in this area are also a key breakthrough for the long-term sustainable development of museum blind box cultural and creative products.

The upper-right quadrant of the matrix represents the strength zone, indicating that consumers place high importance on the content in this area and are satisfied with the current blind box cultural and creative products. This area includes four elements: Aesthetic design (C6), Cultural narrative (C11), Cultural dissemination (C12), and Material (C7). In subsequent design improvement strategies, the current standards should be maintained. Cultural narrative and cultural dissemination can be further deepened through innovative designs inspired by local festivals, historical figures, and landmarks in Macau, continuing the depth of cultural narrative and expanding the reach of cultural dissemination.

The upper-left quadrant of the matrix is the maintenance zone, where consumers perceive the elements in this area as relatively less important in design but have high satisfaction with the current product design. This is an area that does not require significant design improvement strategies. High price (C1) satisfaction indicates a reasonable pricing strategy, and the current pricing evaluation system should be maintained. Consumers are satisfied with the theme series (C8) in the design but do not place high importance on it. We can strengthen promotion in this area, provide a narrative outline, and ensure visual consistency to attract consumers’ attention to the series of products.

### 4.3. Second round of design

Based on the results of the IPA from the first round of design, the updated design underwent a systematic brand deepening process, proposing a unified theme: “Building Macau, Blooming Together.” This theme builds upon the core concepts of “cultural dissemination” and “the integration of art and life” from the Macau Museum’s cultural and creative philosophy. Additionally, by combining the innovative character “Pico” with Macau’s architectural heritage, the blind box products are given a clearer IP setting and richer narrative depth.

In theoretical guidance, the second-round design further strengthened the integrated application of perceived value theory and affective design theory. The social value and emotional value within perceived value theory were concretely manifested through brand affection (C3) and image innovation (C9). The introduction of the IP character “Pico” not only enhanced brand recognition but also stimulated users’ emotional projection and sense of belonging through its anthropomorphic design. The behavioral and reflective layers of affective design are deepened through participatory interaction (C10) and cultural narrative (C11). For instance, story cards guide offline check-ins, extending product usage into real-world scenarios to enhance cultural immersion and meaning-building within the interactive experience.

In the “Building Macau, Blooming Together” theme, ‘Building’ represents Macau’s rich history and cultural heritage, particularly its architectural heritage, which embodies the city’s developmental journey and historical landscape; “Macau” signifies the vibrant city of Macau, expressing its unique geographical and cultural characteristics; “Co-prosperity” conveys the concept of mutual prosperity and cooperation, highlighting Macau’s significant role in national development and its shared goal of prosperity with other regions; “Glory” derives from the lotus flower on Macau’s national flag, symbolizing purity, hope, and the nation’s care and support for the Macau region, embodying the expectation of Macau’s thriving development within the embrace of the motherland.

#### 4.3.1. Brand design.

**Fig 7:**
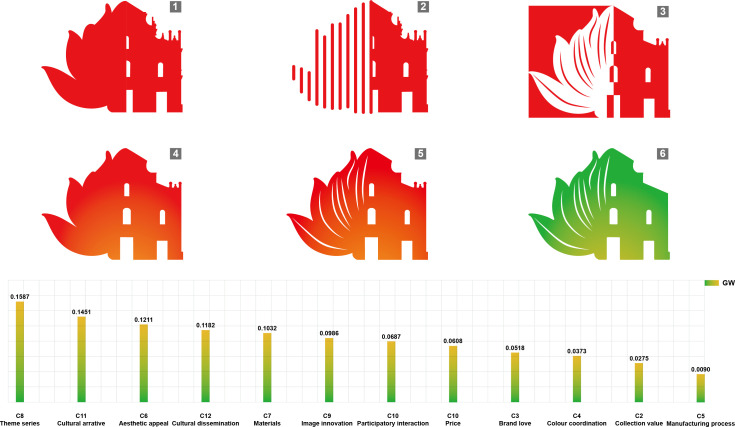
Macau Museum blind box cultural and creative product design first edition IPA results.

The brand logo design is an innovative combination of the Ruins of St. Paul’s and the lotus flower. The Ruins of St. Paul’s (the site of the former St. Paul’s Church) is Macau’s most iconic UNESCO World Heritage Site and the city’s most recognizable symbol, embodying Macau’s rich and diverse cultural heritage; The lotus element in the flag of the Macau Special Administrative Region serves as an official emblem symbolizing prosperity and prosperity, representing Macau’s image of development, openness, and shared prosperity. The architectural silhouette of the Ruins of St. Paul’s Church is seamlessly integrated with the blooming lotus, reflecting the architectural elements incorporated into the product design and symbolizing Macau’s thriving development at the crossroads of Chinese and Western cultures (Fig 7).

#### 4.3.2. Image design.

**Fig 8:**
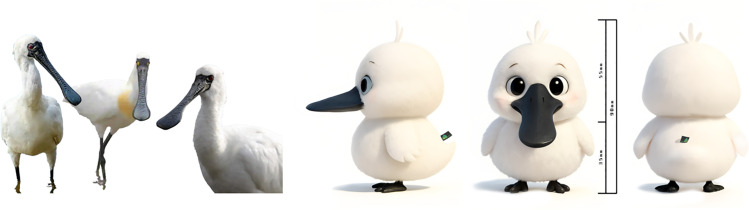
Macau Museum blind box cultural and creative IP image design.

The improved design incorporates the IP character “Pico,” whose prototype is inspired by the critically endangered, black-faced spoonbill, a bird species highly representative of the Macau region. This species symbolizes the coexistence of wetland ecology and urban culture in Macau, as well as the cultural values of peace, spirituality, and ecological harmony. The black-faced spoonbill is a representative species of the “Black Sand Ecological Zone” in Coloane, Macau, and an important component of Macau’s local natural and cultural heritage. Through anthropomorphic design, its image becomes more approachable and adorable, enhancing its communicative appeal; its flat black beak and white feathers serve as high-recognizability features, facilitating consistency in the cartoonized character’s imagery; Additionally, it embodies the ecological dimension of the “art and life connection” concept in Macau Museum’s cultural and creative philosophy, addressing the previous blind spot of “natural absence” in cultural and creative products (Fig 8).

Based on the elements within the lower-left opportunity zone in the first-round design IPA, the “Pico” anthropomorphic character was introduced in image innovation (C9) to tell stories through the IP character, gradually building consumers’ emotional connection to the brand; In terms of collection value (C2), IP-based characters are more conducive to developing limited-edition and limited-quantity cultural and creative products, enhancing their collectible attributes; in terms of materials (C7), plush fabric is used to depict “Pico’s” feathered skin, enhancing the product’s texture.

**Fig 9:**
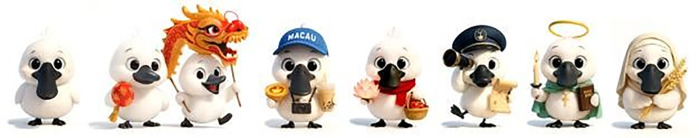
Series image design for the Macau Museum’s blind box cultural and creative product “Pico”.

By integrating Macau’s traditional festivals, cultural architectural features, and tourist preferences, we have created a “Pico” appearance that is contextual, personalized, and offers diverse interactive possibilities. Through multiple role settings and scene derivations, the product combination tells a story (Fig 9).

**Fig 10:**
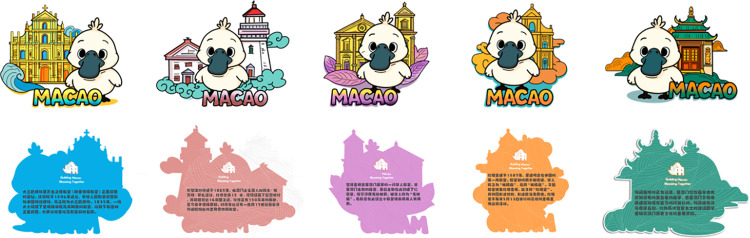
Macau Museum blind box cultural and creative hanging ornament design.

The design is based on the IP character “Pico” and systematically links it with Macau’s iconic landmarks (such as the Ruins of St. Paul’s, A-Ma Temple, Our Lady of the Rosary Church, the East Ocean Lighthouse, and St. Anthony’s Church). Each character’s back features a “cultural storyboard” that introduces the historical background and cultural value of the architecture, achieving a transition from single visual presentation to “information narrative + character-guided exploration.” The cultural narrative (C11) of the product design is reflected in the “building introduction storyboards” paired with the blind boxes, conveying the cultural context of Macau; cultural dissemination (C12) is achieved through the perspective of the “narrator” Pico, presenting culture in an engaging and entertaining manner to enhance its appeal; Image Innovation (C4) involves customizing the IP character’s attire and movements to match different landmarks, creating visual differentiation within the same series; Participatory interactivity (C10) is realized through consumers being guided by story cards to visit physical locations offline, establishing an organic connection between blind box cultural and creative products and real-world tourist destinations (Fig 10).

**Fig 11:**
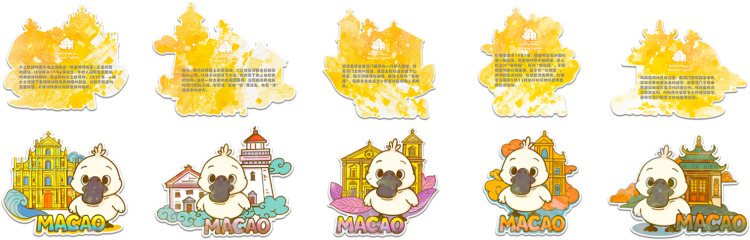
Macau Museum blind box cultural and creative hanging ornament hidden design series.

As a “scarce incentive” within the blind box mechanism, the hidden edition sparks consumers’ curiosity and desire to own the product, driving them to make a purchase. It also manifests as a “special” form of expression in terms of design aesthetics and manufacturing capabilities. In this design, the hidden edition differs significantly from the standard edition in terms of visual language and materials: Color scheme (C4): While retaining the architectural color palette as the base, a unified golden hue is adopted to emphasize “commemoration, honor, and sacredness”; Object materials (C7) feature metallic spray-painted printing, Localized pearlescent materials, and embossed texture coatings; manufacturing processes (C5) employ layered embossed die-cutting techniques and gold foil line treatments to achieve a distinct visual separation from the standard edition (Fig 11).

**Fig 12:**
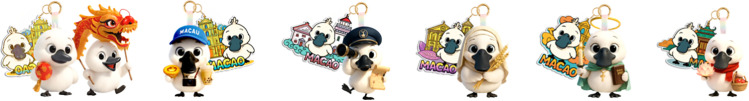
Macau Museum blind box cultural and creative products.

The final blind box cultural and creative products are integrated into keychain pendants, combining the IP character “Pico” with Macau’s iconic buildings and distinctive elements, offering dual value in terms of “city memories” and “character companionship”. The products are divided into five standard variants: Pico is portrayed as a tourist character visiting various Macau landmarks, engaging in activities such as tasting local cuisine, taking photos at popular spots, or holding a map and binoculars while observing the surroundings; one hidden variant: Pico transforms into a dragon dance performer, compatible with all architectural backgrounds, to convey the joyful festive atmosphere that immerses tourists in celebrations across Macau (Fig 12).

Cultural narrative (C11) and Cultural dissemination (C12) are embodied through a thematic connection centered on Macau’s architectural heritage, with IP characters as a supplementary element, emphasizing the emotional interaction between “architecture and IP characters”; Participatory Interaction (C10) features anthropomorphic character designs, incorporates story-based backdrops, and includes removable tags that can be combined with other accessory cards, enhancing flexibility to allow consumers to fully immerse themselves in the scenario; In terms of color coordination (C4), a unified style system is adopted, using classic color schemes from architectural heritage to enhance recognizability; Collection value (C2) is reflected in the differences between hidden edition characters and the uniqueness of object materials. This keychain design represents a significant upgrade in practicality, emotional appeal, and systemization compared to the first version of the architectural signage. It retains the cultural core of Macau Museum’s cultural and creative products while also enhancing user interaction and the market appeal of the product.

#### 4.3.3. Packaging design.

**Fig 13:**
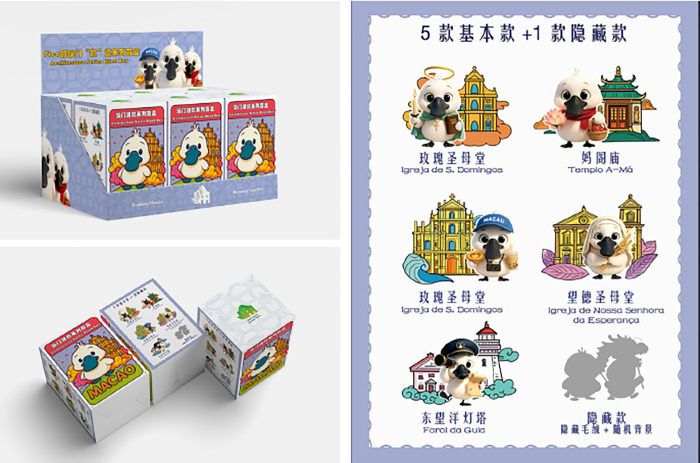
Macau Museum blind box cultural and creative packaging design.

The blind box packaging features a cultural and creative pendant blind box with the “Pico” black-faced spoonbill as the main character, using a unified packaging design, including: an outer box display case; individual box random selection packaging; a product full-style illustration page (right side); through a complete visual system, it achieves the expression of “packaging as culture,” allowing consumers to feel the brand positioning, cultural tone, and visual style before opening the box. Each box contains one plush Pico keychain and one acrylic architectural background panel, with 5 regular variants and 1 hidden variant appearing randomly. The packaging pattern design incorporates Portuguese mosaic tile elements, also showcasing the unique cultural aesthetics of Macau (Fig 13).

The packaging design fully embodies the integration of “cultural content, brand visuals, and product communication.” It not only possesses visual appeal and functionality but also serves as a cultural medium to convey Macau’s diverse cultural environment, enhancing consumers’ cultural and emotional identification, and reflecting multiple key elements of the design criteria.

### 4.4. Consumer satisfaction with the second round of designs

**Fig 14:**
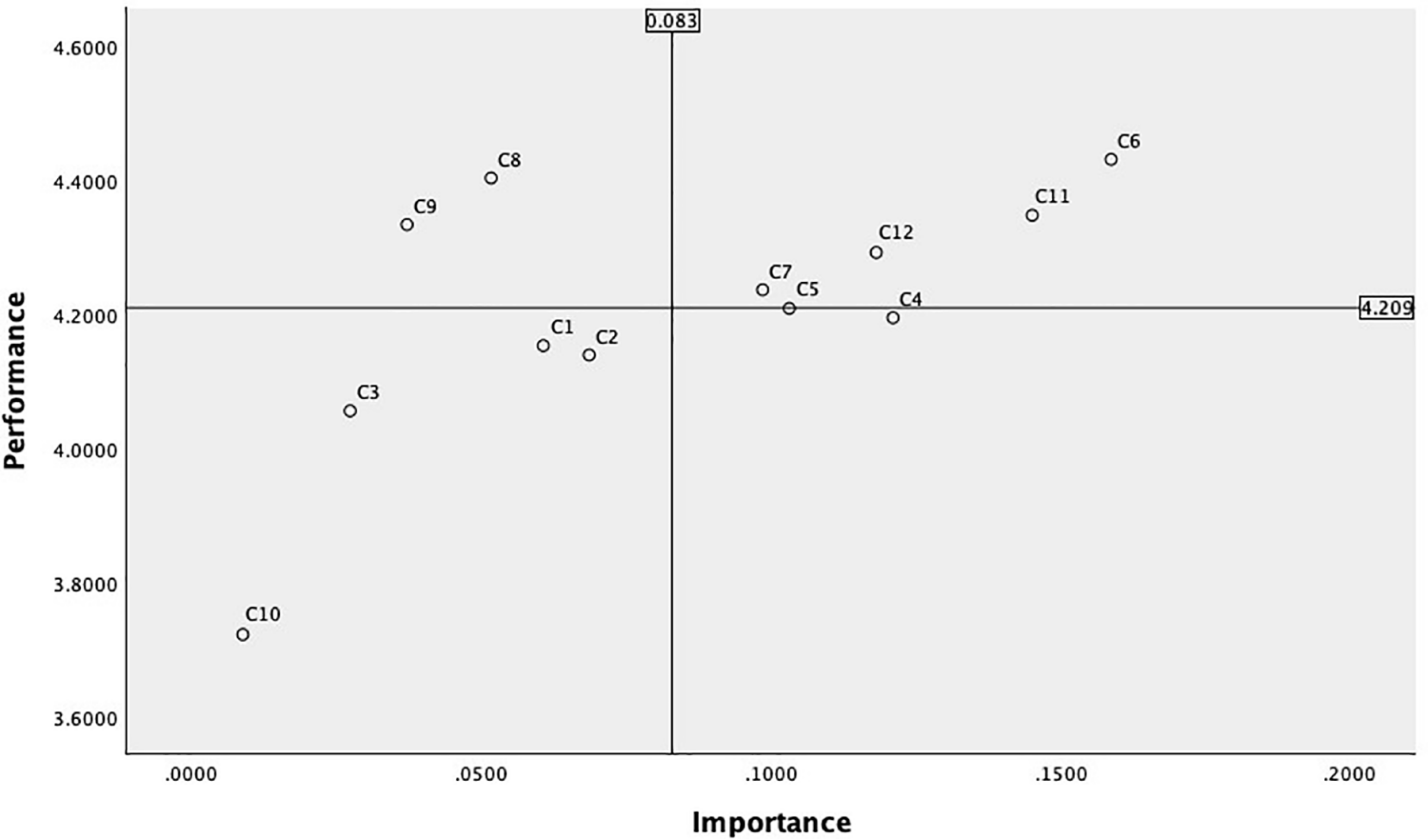
Macau Museum blind box cultural and creative product design second edition IPA results.

The iterative design virtual renderings were converted into a questionnaire and distributed to the 211 participants who effectively completed the questionnaire among the 238 museum cultural and creative product consumers recruited as mentioned in Section 4.2. The iterative design questionnaire continued to employ a 1–5 rating scale. Post-collection analysis yielded a KMO value of 0.923 and a P-value of 0.000. The cumulative variance explained reached 71%, indicating high data reliability(see S6 Table in S2 Appendix). As a result, the research team obtained consumer satisfaction scores for the 12 design elements of the iterated “Pico’s Macau Traces” blind box cultural and creative product [61]. These scores were combined with the AHP design element weights to create a ‘importance’ and “satisfaction” matrix for the 12 elements of the museum blind box cultural and creative product using SPSS 26.0 software. The second IPA matrix uses the weighted average score (0.083) and the satisfaction average score (4.209) as the origin of the coordinate system for constructing the IPA analysis chart (Fig 14).

In the lower right quadrant of the matrix, Color coordination (C4) and Manufacturing process (C5) are still deemed “important but with relatively low satisfaction levels” by consumers in the design. Iterative design has prioritized improvements to these two elements. The Pico keychain has adopted a unified, vibrant color scheme visually, but some designs still exhibit shortcomings in color layering, contrast, and harmony with architectural integration, lacking enhanced expression of high recognizability and cultural charm. In terms of manufacturing processes, the keychain has evolved from the initial acrylic material and card-insert structure to a combination of plush material pendants and acrylic nameplates. Consumers still have expectations regarding the material’s premium feel, detail refinement, and assembly precision. Future improvements should focus on enhancing the three-dimensionality of IP character features, local printing details on tags, and edge sealing treatments.

In the lower-left quadrant of the matrix, the keyring product’s price (C1) continues the “affordable pricing” strategy at 65 MOP, aligning with the entry-level consumption tier for cultural and creative products. With the enhancement of high-quality aesthetics and cultural narratives, consumers’ psychological expectations for the product have quietly risen. Some users even tend to view it as a “collectible decorative item,” so the pricing strategy can transition from a single low price to a tiered structure or a “standard edition + upgraded edition” combination to accommodate the needs of diverse consumer groups; The collection value (C2) system has been preliminarily established, but it needs to be strengthened to enhance a sense of completeness. However, the current collection system still leans toward repetitive patterns and consistent functions, lacking narrative continuity or collection achievement incentives; In terms of brand loyalty (C3), the brand has been preliminarily established, significantly contributing to improved satisfaction with this element; Further efforts can be made to deepen the brand’s core values and expand the variety of content within the brand; Participatory interaction (C10) is beginning to emerge, but the mechanisms remain weak. Currently, product interactivity is primarily limited to visual interactions involving “character and landmark combinations,” lacking a closed-loop mechanism for “user participation, feedback, and growth,” which restricts user engagement and willingness to share.

The top-right quadrant of the matrix represents the strength zone, where aesthetic design, cultural narrative, and dissemination maintain high satisfaction levels. In terms of aesthetic design (C6), new character designs are cuter and more three-dimensional, significantly enhancing visual appeal, fully aligning with the positive feedback of the “strength zone”; Cultural Narrative and Communication (C11, C12) showcase Macau’s diverse cultural dimensions (festivals, cuisine, religion, etc.) through multiple characters, reinforcing the narrative. These three high-importance, high-satisfaction design elements reflect consumers’ strong recognition of the product’s appearance and cultural expression. The iterative keychain series stands out in terms of diverse character designs, refined architectural integration, and synchronized cultural story cards.

The maintenance zone in the upper-left quadrant of the matrix includes the theme series (C8) and Image innovative (C9), which are areas with high consumer satisfaction but low emphasis. The Pico character, an innovative design personifying the black-faced spoonbill, has already formed an initial memory impression in consumers’ minds. It should continue to be maintained and enhanced in terms of image expression, with the appropriate launch of extended merchandise such as series of storage books, display boards, and badges to deepen consumers’ memory of the character. The thematic content of cultural and creative products can be continuously enriched in subsequent designs, such as expanding into food series or festival series, to make this element a standout feature of this blind box-style cultural and creative product and differentiate it from other homogeneous products.

### 4.5. Comparison of two rounds of IPA results

By combining the IPA results obtained from the two designs, consumer satisfaction improved in all aspects of the second design (Table 11). To rigorously validate the effectiveness of design iteration, a paired-sample t-test was conducted on the paired scores from 211 participants. Results indicated that satisfaction scores for all 12 metrics in the second round were significantly higher than those in the first round (p < 0.001). The 95% confidence intervals for all mean differences excluded zero, with effect sizes (Cohen’s d) ranging from 0.51 to 0.87, indicating practical improvements of moderate to large magnitude(Table 11).

**Table 11:** Design two design scheme IPA results.

Criteria	Performance 1 value (1–5)	Performance 2 value (1–5)	Importance value (1–5)	P_1_I	P_2_I	t	p-value	Cohen’s d
C6	3.2059	4.4306	0.1587	0.51	0.70	−7.183	0.000	0.87
C11	3.2059	4.3472	0.1451	0.47	0.63	−6.228	0.000	0.76
C4	3.1176	4.1944	0.1211	0.38	0.51	−6.418	0.000	0.78
C12	3.3529	4.2917	0.1182	0.40	0.51	−5.374	0.000	0.65
C5	3.0147	4.2083	0.1032	0.31	0.43	−6.362	0.000	0.77
C7	3.2647	4.2361	0.0986	0.32	0.42	−5.724	0.000	0.69
C2	3.0735	4.1389	0.0687	0.21	0.28	−5.065	0.000	0.61
C1	3.4853	4.1528	0.0608	0.21	0.25	−4.188	0.000	0.51
C8	3.3676	4.4028	0.0518	0.17	0.23	−5.627	0.000	0.68
C9	3.0882	4.3333	0.0373	0.12	0.16	−6.419	0.000	0.78
C3	3.0735	4.0556	0.0275	0.08	0.11	−5.548	0.000	0.67
C10	2.8382	3.7222	0.0090	0.03	0.03	−4.646	0.000	0.56
Total	–	–	–	3.20	4.27	–	–	–

Price (C1) increased by 0.04, with consumer satisfaction maintaining steady growth, indicating a slight improvement in recognition of price rationality; Collection value (C2) increased by 0.07, with the introduction of hidden editions enhancing logical appeal, attracting consumer attention to the product’s collectible attributes; Brand loyalty (C3) increased by 0.03, as consumers have begun to recognize the Pico IP image, though emotional attachment is still in the cultivation phase; Color coordination (C4) increased by 0.13, indicating significant improvements in visual language and more reasonable integration of cultural colors; Manufacturing process (C5) increased by 0.12, reflecting enhancements in craftsmanship (materials, texture, structure) and optimized details; Aesthetic appeal (C6) increased by 0.19, highlighting the prominence of the exterior design, with the Pico character design gaining favor; Materials (C7) improved by 0.1, indicating a more textured material choice that better aligns with the cultural positioning of the creative product; Theme series (C8) increased by 0.06, with architecture and characters corresponding one-to-one to create a sense of series, and establishing distinct design differentiation; Image innovation (C9) increased by 0.04, with multi-character style differentiation design, and preliminary establishment of innovative expression; Participatory Interaction (C10) did not increase, indicating it remains a design weakness, with interactive gameplay (such as scanning codes, check-ins) yet to be established; Cultural narrative (C11) increased by 0.16, with the design of architectural story cards and contextual explanation boards reflecting the cultural narrative within the product, enhancing users’ sense of cultural immersion; Cultural dissemination (C12) increased by 0.11, with the influence of distinctive packaging design and IP character systems contributing to the dissemination of Macau’s unique culture through this blind box product.

**Fig 15:**
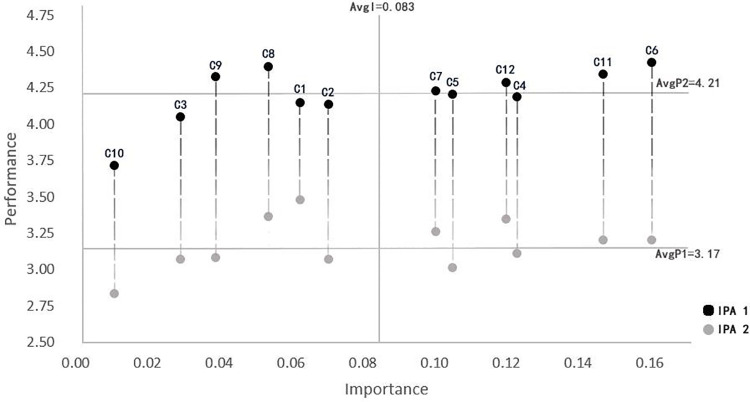
Comparison of IPA results for two design versions.

After design updates, elements with significant growth (growth ≥ +0.10): Aesthetic appeal (C6), Cultural narrative (C11), Color coordination (C4), and Manufacturing process (C5), indicating that the design has achieved notable results in visual and cultural presentation dimensions; elements with steady improvement (+0.05 to +0.10): Collectible value (C2), Materials (C7), and Cultural dissemination (C12); Slowly improving elements (<+0.05): Price (C1), Brand Loyalty (C3), Image innovative (C9), Theme series (C8), indicating potential for further development in brand operations, character interaction, and narrative; elements that did not improve: Participatory interaction (C10) is a key breakthrough area for the next round of design optimization [62] (Fig 15).

The second design iteration achieved a comprehensive upgrade across core design dimensions such as visual identity, aesthetic harmony, and cultural narrative. The overall consumer satisfaction index increased from 3.20 to 4.27 (+1.07), demonstrating that the “Pico’s Macau Heritage” design system has been preliminarily established. Going forward, it is recommended to focus on interactive mechanisms (C10) and brand emotional development (C3) as strategic breakthrough points to build a closed-loop cultural and creative IP ecosystem that is participatory, collectible, and shareable.

## 5. Discussion

### 5.1. Design improvement direction

Compared to the architectural signpost blind box, the “Pico’s Macau Landmarks” pendant blind box has been optimized and improved in multiple dimensions, with enhanced stability across all elements [63]. In the initial product design, the color coordination (C4) and manufacturing process (C5) in the repair area required significant improvement, but after iteration, the IPA performance values have shown a noticeable increase. In the advantage areas, elements such as aesthetic design, material quality, and cultural narrative continue to maintain high satisfaction levels, indicating that the product possesses strong competitiveness in both design and quality. In the second iteration of the pendant blind box design, the brand and IP character Pico were incorporated, with a focus on brand loyalty (C3) and image innovation (C9), thereby enhancing consumers’ brand awareness and image recall of museum cultural and creative products.

By focusing on cultural depth and craftsmanship upgrades, consumer perception was enhanced. The improved numerical values are more concentrated (mostly within the 3.72–4.43 range), reflecting more balanced decision-making execution and improvements in weak areas, though ongoing attention is needed to optimize interactivity.

In the future, a “Pico City Tour” map could be created, with characters corresponding to Macau landmarks, encouraging “check-in collection”; peripheral products (hats, backpacks, etc.) could be developed in conjunction with the characters to enhance brand influence; and online content such as Pico diaries, festival comics, and interactive audio stories could be developed across multiple internet platforms to enhance IP brand fan loyalty.

### 5.2. Comparison of existing scales

This study preliminarily identified factors such as “product scent,” “logistics speed,” and “packaging quality” through online review analysis. These elements have not been systematically incorporated into existing museum cultural and creative product or blind box design scales. For instance, the blind box product characteristic scale proposed by Zhan & Xiong (2024) suggests that uncertainty, sociality, aesthetics, and fun positively influence consumer purchase intent [64]. Hu (2024) identifies two distinct blind box sales scenarios: online and offline [30]. Offline channels typically offer superior service experiences, while online channels enhance the probability of purchasing popular products. Within the online shopping context, the newly proposed “logistics speed” factor in this study represents an aspect not addressed by Hu (2024) [30]. Xu et al. (2025) constructed a scale measuring uncertainty, scarcity, functionality, aesthetics, and symbolic meaning [65]. However, this scale primarily focuses on consumers’ emotional experiences, with limited discussion on design decisions. Reviewing other literature reveals fragmented focus on attributes like surprise experiences, collecting desires, social interactions, IP imagery, and aesthetic value. However, “product scent” relates to sensory experiences, “logistics speed” connects to service efficiency, and “packaging quality” impacts first impressions and practicality—all crucial components of the consumer experience chain. Although some elements failed to meet the consensus threshold in FDM expert screening—potentially because they lean more toward product delivery and usage scenarios rather than core design principles—their repeated mention in consumer reviews indicates they potentially influence purchasing decisions and satisfaction. This suggests that existing blind box cultural and creative design scales may need to expand their measurement dimensions to encompass broader consumption contexts. Future research could further examine the weighting of these factors across different cultural markets to enhance the integrity of design evaluation systems.

### 5.3. Correlation analysis between factors

**Fig 16:**
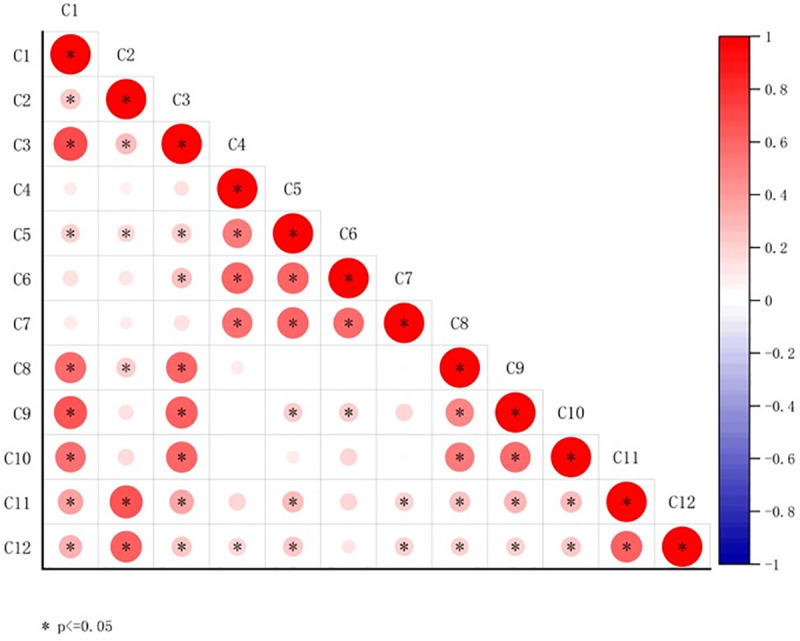
Correlation coefficient heat map.

An analysis of consumer satisfaction scores for blind box products from the second IPA questionnaire revealed a certain correlation between different factors. To investigate which specific factors were interrelated, a correlation analysis of the data was conducted. Kendall tau_b coefficient range: [−1, 1]. The closer to ±1, the stronger the correlation [66]. Significance notation: * indicates significance at the 0.05 level (two-tailed); ** indicates significance at the 0.01 level (two-tailed) [67] (Fig 16).

The correlation coefficient heatmap illustrates the relationships among the 12 dimensions in the cultural and creative industry: C1 (Price) exhibits varying degrees of association with other dimensions, without forming distinct clusters of strong correlations; C2 (Collectible value) shows moderate associations with certain cultural and quality dimensions but does not form prominent strong correlations; C3 (Brand loyalty) has mild associations with most dimensions and relies more on comprehensive factors; C4 (Color coordination) is a relatively independent dimension, showing weak correlations with most dimensions such as C1–C3 and C6–C9, and even exhibiting a weak negative correlation trend with C8 (Theme series); C5 (manufacturing process) forms a strong positive correlation cluster with C6 (Aesthetic appeal) and C7 (Materials), reflecting the synergistic nature of product quality; C6 (Aesthetic Appeal) has strong correlations with C5 and C7, while its associations with other dimensions are moderate; C7 (Materials) has strong correlations with C5 and C6, but its influence on other dimensions is relatively indirect; C8 (Theme series) has weak associations with most dimensions and exhibits notable independence; C9 (Image innovation) has relatively balanced associations with other dimensions, without showing extreme strengths or weaknesses; C10 (Participatory Interaction) forms a significant strong positive correlation with C11 (Cultural narrative) and C12 (Cultural communication), reflecting the interconnectedness of cultural experience and communication; C11 (Cultural Narrative) is strongly correlated with C10 and C12, and also has some association with product hard quality dimensions; C12 (Cultural Communication) serves as a key node in the synergy of cultural dimensions through its strong correlations with C10 and C11. Overall, the dimensions are primarily positively correlated, with distinct “hard quality clusters” (C5–C7) and “cultural communication clusters” (C10–C12). The independence of C4 (Color coordination) and the weak correlations of some dimensions provide data-driven insights for differentiation strategies in cultural and creative products.

## 6. Conclusion

This study employs inductive reasoning, case analysis, and the FDM to screen and identify criteria influencing museum blind box-style cultural and creative products. It references the AHP hierarchical analysis method to determine the weighting ratios of each criterion, and conducts two design practices based on Macau museum blind box-style cultural and creative products. The study adopts a consumer-centric perspective, and its findings provide theoretical guidance and optimization recommendations for museum decision-makers in the development and design of blind box-style cultural and creative products:

Emphasize artistic aesthetics: Artistic aesthetics constitute the core competitiveness of the product. It is essential to create visually appealing and high-quality products through multiple dimensions such as form, color, and craftsmanship to stimulate consumer purchasing interest.

Emphasize cultural connotations: As cultural carriers, museum blind box-style cultural and creative products should convey the unique cultural value of the museum through storytelling and interactivity, strengthening emotional connections with consumers.

Balance basic needs: While ensuring artistic and cultural value, control costs, lower consumption barriers, and develop series-based blind box products. By continuously introducing innovative products, long-term appeal can be enhanced.

This study focuses on consumers’ purchasing intentions for museum blind box-style cultural and creative products. Future research could expand to explore consumer preferences for blind box-style cultural and creative products across different regions and cultural contexts (e.g., acceptance of traditional cultural symbols, price sensitivity, etc.), comparing preferences between first-tier cities and lower-tier markets, or between domestic and international tourists. Additionally, future designs could explore the integration of AR and VR technologies to develop digital blind boxes (virtual artifact unlocking), investigating whether the fusion of science and technology with cultural and creative products enhances consumers’ purchasing intentions.

### 6.1. Limitation

Despite two rounds of design practice and the collection of IPA data for analysis, there are still limitations: the sample size of the survey is relatively small, and there is an imbalance in the proportion of tourists versus local residents and younger versus older groups, which may cause the data results to be more biased toward a specific consumer group (such as young tourists or students). The adaptability of the findings for the general population still requires further validation.

Participatory interaction (C10) showed limited growth in the two rounds of IPA results. While it has a positive correlation with variables such as brand loyalty, users have a weak understanding of the interaction mechanisms. This may be because the interaction forms remain at the design intent level and have not been effectively implemented or experienced before the questionnaire, leading to lower evaluations; Cultural dissemination (C12) and Image innovative (C9) showed no significant correlation in the correlation analysis, indicating that while the design incorporates cultural content, there is a lack of integration between dissemination methods and design expression. The current product remains focused on “static narrative” and lacks dynamic, contextual, or participatory dissemination forms.

The two design iteration cycles were relatively concentrated, possibly failing to fully incorporate market feedback to form a more mature and stable design strategy. Users’ affection for the Pico character’s image is still in its early stages of development, making it difficult to form deep cultural identity in a short period of time. All variable correlations are based on Kendall’s rank correlation analysis, indicating “statistical correlation,” but this does not imply a definitive causal relationship between variables. Design optimization still requires cross-validation through methods such as user interviews, observations, and experimental testing.

## Supporting information

S1 Appendix Standardized coding manual. (DOCX)

S2 Appendix Coder reliability and three CFA report values. (DOCX)

S3 Appendix Results of two rounds of FDM. (XLSX)

S4 Appendix Summary calculation of all AHP results. (XLSX)
