# Supplementary material for: “Opening the Box to Explore the Contents”: A study on the design elements of museum cultural and creative blind boxes based on consumer preferences – Taking the Macao Museum as an example
Source: PLoS One. 2026 Mar 6;21(3):e0344422. doi: 10.1371/journal.pone.0344422 (PMC12965585; doi:10.1371/journal.pone.0344422)
Supplement: S1 Appendix — (DOCX) [file pone.0344422.s001.docx]

| **Code (Criteria)** | **Operational Definition** | **Inclusion Criteria** | **Exclusion Criteria** | **Anchor Examples** |
| --- | --- | --- | --- | --- |
| **E1 Price** | Consumers' assessment of product value for money and their acceptable price range. | Mentions of "expensive", "cheap", "worth it", "cost-effective", specific prices, or discounts. | General comments on value not related to monetary cost (e.g., "valuable experience"). | "It's cheaper than in stores."  "I spent 90 yuan... if the cost-effectiveness could be improved." |
| **E2 Collection value** | The desire to collect the series and the surprise experience brought by the unboxing process. | Mentions of "collecting", "full set", "hidden edition", "surprise", or "regret" regarding specific draws. | Comments on the product's utility or general appearance without reference to collecting behavior. | "I really wanted to win the ginger and scallion chicken... I’ll definitely try again next time!" |
| **E3 Brand loyalty** | The degree to which consumers like a specific museum brand or its thematic culture. | Mentions of being a "fan" of the museum, visiting the museum, or trust in the museum's IP. | Liking the product solely for its looks without referencing the museum brand context. | "As a die-hard fan of the Guangdong Provincial Museum, how could I not support..." |
| **E4 Social communication** | Sharing behavior with friends/social media, including gifting and recommendations. | Mentions of "gift", "giving to friends", "sharing", "posting photos", or "recommending". | Solitary usage or enjoyment without social interaction mentioned. | "It was a gift from a friend."  "It's also suitable for children to play with." |
| **E5 Logistics speed** (New) | The time efficiency from purchase to receipt of the product. | Mentions of "shipping speed", "delivery time", "fast arrival", "slow delivery". | Comments on the packaging condition (see E9) rather than speed. | "The shipping speed was very fast."  "I received it in just a few days." |
| **E6 Practical functions** | The product's utility and functionality in everyday life beyond decoration. | Mentions of usage (e.g., "fridge magnet", "bookmark", "desktop ornament") or lack of utility. | Purely aesthetic appreciation without mentioning specific usage scenarios. | "Can be used as a magnet..." |
| **E7 Color coordination** | The harmony, accuracy, or appeal of the product's color scheme. | Mentions of "color", "paint", "vibrant", "faded", or color accuracy compared to originals. | General comments on "looking good" (see E11) without specific reference to color. | "The color is almost identical to the other two."  "This color is super cute." |
| **E8 Product odor** (New) | Any scent (pleasant or unpleasant) detected upon unboxing. | Mentions of "smell", "odor", "scent", "fumes", "airing out". | Comments on visual defects or tactile feel. | "There is a strong odor... similar to the smell of a cheap, patterned phone case." |
| **E9 Packaging quality** (New) | The physical condition and design quality of the external packaging upon arrival. | Mentions of "box", "package", "damaged", "intact", "protection". | Comments on the product inside the box (unless damage transferred). | "The packaging was well done, with no damage."  "The package was quite heavy, feeling substantial." |
| **E10 Manufacturing craftsmanship** | The technical quality, finish, and detail execution of the physical product. | Mentions of "workmanship", "details", "flaws", "defects", "paint job", "texture". | General aesthetic praise (see E11) or material composition (see E12) unless related to build quality. | "The craftsmanship is quite intricate, even the patterns are detailed."  "There is a dark spot on the left wing that cannot be wiped off." |
| **E11 Aesthetic appeal** | The overall beauty, visual refinement, and attractiveness of the design. | Mentions of "beautiful", "cute", "good-looking", "ugly", "design style". | Specific comments on color (E7) or innovation (E14) are coded separately if precise. | "It looks very beautiful on my desk."] |
| **E12 Materials** | The tactile and visual qualities of the materials used (e.g., plastic, metal, ceramic). | Mentions of "texture", "heavy", "light", "plastic feel", "ceramic", "metal". | Comments on the assembly or flaws (E10). | "It’s heavier than I expected."  "The bronze artifact... felt quite substantial and had a nice texture." |
| **E13 Theme series** | Coherence and continuity across the product line or set. | Mentions of "series", "set", "whole collection", or thematic consistency. | Single product evaluation without reference to the broader series. | “Based on millennia-old artifacts such as the bronze standing figure and the elongated-eye mask” |
| **E14 Image innovation** | Creativity in character design, IP adaptation, or novel expression forms. | Mentions of "creative", "unique", "novel idea", "interesting design". | Standard descriptions of the artifact without noting creativity. | "The design is truly creative."  "The idea of combining archaeological excavation with blind boxes is quite creative." |
| **E15 Participatory interaction** | The degree of physical engagement or interactivity required/offered by the product. | Mentions of "digging", "assembling", "DIY", "playing", "interaction". | Passive observation or static display. | "Using the shovel and brush provided to dig it out was very interactive." |
| **E16 Cultural narrative** | The product's ability to convey specific stories, history, or knowledge. | Mentions of "history", "learning", "story", "education", "meaning". | General mentions of "culture" without specific narrative content. | "Can be used to teach them about history."  "Open the door to good fortune" (symbolic meaning). |
| **E17 Cultural dissemination** | The product's potential to promote awareness or expand cultural influence. | Mentions of "promoting culture", "souvenir", "representing [Place/Museum]". | Personal enjoyment of the story (E16) without the broader dissemination aspect. | "How could I not support the cultural and creative products..." |
