# Supplementary material for: “Opening the Box to Explore the Contents”: A study on the design elements of museum cultural and creative blind boxes based on consumer preferences – Taking the Macao Museum as an example
Source: PLoS One. 2026 Mar 6;21(3):e0344422. doi: 10.1371/journal.pone.0344422 (PMC12965585; doi:10.1371/journal.pone.0344422)
Supplement: S2 Appendix — (DOCX) [file pone.0344422.s002.docx]

S1 Table. Holsti's Coefficient Reliability

|  | Consistent encoding number（N=864） | Degree of mutual consent | Holsti's Coefficient |
| --- | --- | --- | --- |
| Coders #1 and #2 have the same number | 724 | 0.8379 | 0.9429 |
| Coders #2 and #3 have the same number | 732 | 0.8472 |  |
| Coders #1 and #3 have the same number | 738 | 0.8541 |  |

S2 Table. Model Fit Verification

| Indicator | Test Results |
| --- | --- |
| CMIN/DF | 1.933 |
| RMSEA | 0.067 |
| SRMR | .0049 |
| TLI | 0.971 |
| CFI | 0.977 |

S3 Table. Convergent Validity and Combinatorial Reliability Tests

| Path | Estimate | AVE | CR |
| --- | --- | --- | --- |
| C10Participatory interaction<---Product value and experience | 0.857 | 0.713 | 0.925 |
| C9Image innovation<---Product value and experience | 0.826 |  |  |
| C8Theme series<---Product value and experience | 0.81 |  |  |
| C3 Brand loyalty <---Product value and experience | 0.832 |  |  |
| C1Price<---Product value and experience | 0.895 |  |  |
| C7Materials<---Design aesthetics and craftsmanship | 0.775 | 0.73 | 0.915 |
| C6Aesthetic appeal<---Design aesthetics and craftsmanship | 0.911 |  |  |
| C5manufacturing craftsmanship<---Design aesthetics and craftsmanship | 0.841 |  |  |
| C4Color coordination<---Design aesthetics and craftsmanship | 0.884 |  |  |
| C12Cultural dissemination<---Cultural drivers and identity | 0.914 | 0.861 | 0.949 |

S4 Table. HTMT Results

|  | Product value and experience | Design aesthetics and craftsmanship | Cultural drivers and identity |
| --- | --- | --- | --- |
| Product value and experience |  |  |  |
| Design aesthetics and craftsmanship | 0.452 |  |  |
| Cultural drivers and identity | 0.345 | 0.34 |  |

S5 Table. First IPA Questionnaire Results

| First IPA Questionnaire | |
| --- | --- |
| Criteria | Factor load factor |
| C1 Price | 0.685 |
| C3 Brand loyalty | 0.884 |
| C8 Theme series | 0.863 |
| C9 Image innovation | 0.865 |
| C10 Participatory interaction | 0.898 |
| C4 Color coordination | 0.781 |
| C5 manufacturing craftsmanship | 0.849 |
| C6 Aesthetic appeal | 0.818 |
| C7 Materials | 0.808 |
| C2 Collection value | 0.775 |
| C11 Cultural narrative | 0.816 |
| C12 Cultural dissemination | 0.757 |
| Cumulative variance interpretation rate % (after rotation) | 67.000% |
| KMO Value | 0.947 |
| Bart spherical value | 645.011 |
| df | 164 |
| P Value | 0.000 |

S6 Table. Second IPA Questionnaire Results

| Second IPA Questionnaire | |
| --- | --- |
| Criteria | Factor load factor |
| C1 Price | 0.723 |
| C3 Brand loyalty | 0.844 |
| C8 Theme series | 0.712 |
| C9 Image innovation | 0.791 |
| C10 Participatory interaction | 0.668 |
| C4 Color coordination | 0.755 |
| C5 manufacturing craftsmanship | 0.727 |
| C6 Aesthetic appeal | 0.839 |
| C7 Materials | 0.734 |
| C2 Collection value | 0.832 |
| C11 Cultural narrative | 0.829 |
| C12 Cultural dissemination | 0.782 |
| Cumulative variance interpretation rate % (after rotation) | 0.71 |
| KMO Value | 0.923 |
| Bart spherical value | 682.481 |
| df | 158 |
| P Value | 0.000 |
